# Deep-intronic *ABCA4* variants explain missing heritability in Stargardt disease and allow correction of splice defects by antisense oligonucleotides

**DOI:** 10.1038/s41436-018-0414-9

**Published:** 2019-01-15

**Authors:** Riccardo Sangermano, Alejandro Garanto, Mubeen Khan, Esmee H. Runhart, Miriam Bauwens, Nathalie M. Bax, L. Ingeborgh van den Born, Muhammad Imran Khan, Stéphanie S. Cornelis, Joke B. G. M. Verheij, Jan-Willem R. Pott, Alberta A. H. J. Thiadens, Caroline C. W. Klaver, Bernard Puech, Isabelle Meunier, Sarah Naessens, Gavin Arno, Ana Fakin, Keren J. Carss, F. Lucy Raymond, Andrew R. Webster, Claire-Marie Dhaenens, Heidi Stöhr, Felix Grassmann, Bernhard H. F. Weber, Carel B. Hoyng, Elfride De Baere, Silvia Albert, Rob W. J. Collin, Frans P. M. Cremers

**Affiliations:** 1 Department of Human Genetics, Radboud University Medical Center, Nijmegen, The Netherlands; 2 Radboud Institute for Molecular Life Sciences, Radboud University Medical Center, Nijmegen, The Netherlands; 3 Donders Institute for Brain, Cognition and Behaviour, Radboud University Medical Center, Nijmegen, The Netherlands; 4 Department of Ophthalmology, Radboud University Medical Center, Nijmegen, The Netherlands; 5 Center for Medical Genetics, Ghent University and Ghent University Hospital, Ghent, Belgium; 6 The Rotterdam Eye Hospital and the Rotterdam Ophthalmic Institute, Rotterdam, The Netherlands; 7 Department of Medical Genetics, University Medical Center Groningen, University of Groningen, Groningen, The Netherlands; 8 Department of Ophthalmology, University Medical Center Groningen, University of Groningen, Groningen, The Netherlands; 9 Department of Ophthalmology, Erasmus Medical Center, Rotterdam, The Netherlands; 10 Service d′Exploration de la Vision CHU, Lille, France; 11 Institute for Neurosciences of Montpellier INSERM U1051, University of Montpellier, Montpellier, France; 12 Centre d′Etude du Polymorphisme Humain, Fondation Jean Dausset, Paris, France; 13 UCL Institute of Ophthalmology, London, UK; 14 Moorfields Eye Hospital, London, UK; 15 Department of Haematology, University of Cambridge, Cambridge, UK; 16 NIHR BioResource, Cambridge University Hospitals NHS Foundation Trust, Cambridge Biomedical Campus, Cambridge, UK; 17 Department of Medical Genetics, Cambridge Institute for Medical Research, University of Cambridge, Cambridge, UK; 18 Univ. Lille, Inserm UMR-S 1172, CHU Lille, Biochemistry and Molecular Biology Department - UF Génopathies, Lille, France; 19 Institut für Humangenetik, Universität Regensburg, Regensburg, Germany; 20 Department of Medical Epidemiology and Biostatistics, Karolinska Institutet, Stockholm, Sweden

**Keywords:** *ABCA4*, antisense oligonucleotide, deep-intronic variant, missing heritability, Stargardt disease

## Abstract

**Purpose:**

Using exome sequencing, the underlying variants in many persons with autosomal recessive diseases remain undetected. We explored autosomal recessive Stargardt disease (STGD1) as a model to identify the missing heritability.

**Methods:**

Sequencing of *ABCA4* was performed in 8 STGD1 cases with one variant and p.Asn1868Ile in *trans*, 25 cases with one variant, and 3 cases with no *ABCA4* variant. The effect of intronic variants was analyzed using in vitro splice assays in HEK293T cells and patient-derived fibroblasts. Antisense oligonucleotides were used to correct splice defects.

**Results:**

In 24 of the probands (67%), one known and five novel deep-intronic variants were found. The five novel variants resulted in messenger RNA pseudoexon inclusions, due to strengthening of cryptic splice sites or by disrupting a splicing silencer motif. Variant c.769-784C>T showed partial insertion of a pseudoexon and was found in *cis* with c.5603A>T (p.Asn1868Ile), so its causal role could not be fully established. Variant c.4253+43G>A resulted in partial skipping of exon 28. Remarkably, antisense oligonucleotides targeting the aberrant splice processes resulted in (partial) correction of all splicing defects.

**Conclusion:**

Our data demonstrate the importance of assessing noncoding variants in genetic diseases, and show the great potential of splice modulation therapy for deep-intronic variants.

## INTRODUCTION

In the past decade, next-generation sequencing (NGS) has enabled a rapid identification of novel disease genes,^1–3^ and transformed molecular diagnostic testing.^4–6^ Thus far, the emphasis was on coding and flanking splice site sequences that harbor the majority of genetic defects. Herewith, the underlying defects could be identified in ~50% of individuals with genetically heterogeneous diseases, including inherited retinal diseases (IRDs).^7–9^ Genome sequencing can identify the majority of noncoding sequence variants,^1^ but it will be very challenging to pinpoint the causal variants in the absence of assays that reveal the resulting messenger RNA (mRNA) and/or protein defects.

For diseases that are due to variants in a single gene, the yield of variants generally exceeds 50%. This is true for Stargardt disease (STGD1) (MIM 248200), which is caused by pathogenic variants in the gene encoding the adenosine triphosphate (ATP) binding cassette type A4 (*ABCA4*) (MIM 601691)^10^ as biallelic variants can be found in the majority of the cases^11^ and in a smaller proportion of other IRD subtypes.^12–14^ In ~30% of STGD1 cases, however, the expected second *ABCA4* variant was lacking. A frequent coding variant, p.Asn1868Ile, was present in ~10% of STGD1 cases who had a severe pathogenic variant in *trans* and who generally showed late-onset disease.^15,16^ The remaining pathogenic variants in *ABCA4* rarely consist of heterozygous deletions,^17–19^ whereas deep-intronic *ABCA4* variants also account for some of the missing heritability in STGD1.^11,17,20,21^ By employing STGD1-derived keratinocytes, it was shown that some of these variants strengthen cryptic splice sites or create new splice sites, ultimately resulting in pseudoexon (PE) inclusions.^21^ Using STGD1-derived photoreceptor precursor cells (PPCs), we recently showed that two intronic variants in *ABCA4* (c.4539+2001G>A and c.4539+2028C>T) strengthen exonic splice enhancer elements (ESEs), resulting in the inclusion of a 345-nt PE.^22^

PE inclusions due to deep-intronic variants are attractive targets for antisense oligonucleotide (AON)-based splicing correction.^23^ In the field of IRDs, these molecules have shown therapeutic potential for deep-intronic pathogenic variants in *CEP290,*^24–27^ *USH2A*,^28^ or *OPA1,*^29^ and a phase 1/2 clinical trial using AONs to treat *CEP290*-associated Leber congenital amaurosis is ongoing (NCT03140969).

In this study, we employed STGD1 as a model to assess the challenges to identify pathogenic noncoding variants. We sequenced the *ABCA4* locus in 36 STGD1 individuals with one *ABCA4* variant and p.Asn1868Ile in *trans* (*n* = 8), one *ABCA4* variant (*n* = 25), or no (*n* = 3) *ABCA4* variants. Selected noncoding variants were tested by in vitro splice assays or using STGD1-derived fibroblasts. Subsequently, several AONs were designed and successfully applied to redirect erroneous splicing.

## MATERIALS AND METHODS

### Samples

Written informed consent was obtained prior to participation in the study, which adhered to the Declaration of Helsinki. This study was approved by the Medical Ethical Committee 2010-359 (Protocol nr. 2009-32; NL nr. 34152.078.10) and the Commissie Mensgebonden Onderzoek Arnhem-Nijmegen (Dossier no. 2015-1543; dossier code sRP4h). More information regarding the proband cohort, and details of the materials and methods used, are provided in the Supplementary Material.

### *ABCA4* sequence analysis and selection of candidate splice variants

Detailed information of the selection of candidate splice variants and inclusion criteria is provided in the Supplementary Material.

### Midigene-based splice assay

The effect of 11 noncoding variants was assessed by midigene-based splicing assays employing seven wild-type (WT) BA clones described previously^30^ and the newly designed BA30. WT and mutant constructs were transfected in HEK293T cells and the extracted total RNA was subjected to reverse transcription polymerase chain reaction (RT-PCR) (Table S1) as described previously.^30^

### Single-molecule molecular inversion probe–based sequence analysis of intronic *ABCA4* regions

To test the occurrence of five intronic *ABCA4* variants (c.769-784C>T, c.859-506G>C, c.4253+43G>A, c.4539+1100A>G, c.4539+1106C>T) in 412 genetically unsolved STGD1 cases from France (*n* = 224) or Germany (*n* = 188), we designed single-molecule molecular inversion probes (smMIPs) based on a previously developed cost-effective sequencing protocol.^31^ The variant calling and annotation was performed using an in-house pipeline. The number of single-molecule consensus reads ranged from 14 to 1587, with an average coverage of 489x.

### Antisense oligonucleotides

For each deep-intronic variant that caused PE inclusion, three AONs were designed (Table S2). The design of the therapeutic molecules was performed as described elsewhere.^32^ Oligonucleotides had a phosphorothioate backbone with a 2-*O*-methyl sugar modification (2OMe/PS) and were synthesized by Eurogentec. The oligonucleotides were resuspended in phosphate-buffered saline (PBS) and used at a concentration of 0.5 µM.

### In vitro rescue studies in HEK293T cells using midigenes and AONs

HEK293T cells were transfected with either the wild-type or the mutant construct. Twenty-four hours posttransfection, each well was subdivided in five wells to be transfected with the respective AON, the sense oligonucleotide (SON), or left untransfected (NT). Forty-eight hours post-AON delivery, cells were harvested and RNA analysis was performed by RT-PCR. All experiments were performed in two independent replicates. Primer sequences can be found in Table S1.

### Rescue studies using antisense oligonucleotides in fibroblasts

Fibroblast cells were transfected with each of the antisense oligonucleotides, or transfected with empty liposomes (NT). Forty-four hours posttransfection, medium was removed and medium containing cycloheximide (CHX) was added to block nonsense-mediated decay (NMD). Four hours later, cells were harvested and subjected to RNA analysis by RT-PCR. All experiments were performed in two independent replicates. Primer sequences are indicated in Table S1.

### Capillary analysis

To quantify the ratios between correct and aberrant transcripts we loaded the RT-PCR samples onto a Fragment Analyzer Auto Capillary Electrophoresis System (Advanced Analytical Technologies, Inc.). Analysis of the peaks was performed using the corrected peak area parameter and only the peaks corresponding to the expected bands were taken into account.

## RESULTS

### Identification and selection of deep-intronic variants for splicing assay

Haloplex sequencing of eight STGD1 cases carrying one causal *ABCA4* variant and p.Asn1868Ile in *trans*, 22 STGD1 cases with only one, and two cases carrying no *ABCA4* variant, yielded 220 rare *ABCA4* variants (allele frequency [AF] ≤0.01 in control individuals), consisting of 85 independent novel variants (Table S3). In the four British STGD1 cases with one (*n* = 3) or no (*n* = 1) *ABCA4* variant analyzed by genome sequencing, eight novel rare (AF ≤0.01) independent *ABCA4* variants remained (Table S3).

Eleven noncoding variants were selected for splice assays (Table S4). Seven of these (c.768+7329A>G, c.858+526T>G, c.859-506G>C, c.1555-5008C>T, c.4539+1100A>G, c.4539+1106C>T, c.6148-421T>C) were selected as they were located at cryptic splice sites or created a new putative splice site with a relative strength of at least 75% of the maximal score in two of five algorithms, and showed an increased splice prediction score of at least 2% (Table S4). Two variants, c.769-784C>T and c.4253+43G>A, belonging to three alleles (c.[769-784C>T; 5603A>T], c.[4253+43G>A; 6006-609T>A], c.[4253+43G>A; 5603A>T]), were enriched among the monoallelic cases, i.e., in 7 and 9 of 36 probands, respectively. The c.4253+43G>A variant was reported previously.^33^ Finally, the c.1937+435C>G variant was selected because it was located in the genomic sequence of an alternate exon in intron 13 (Chr1: 94,527,737–94,527,644).^21^

### Four novel deep-intronic variants result in PE generation through the strengthening of splice sites

**Fig. 1:**
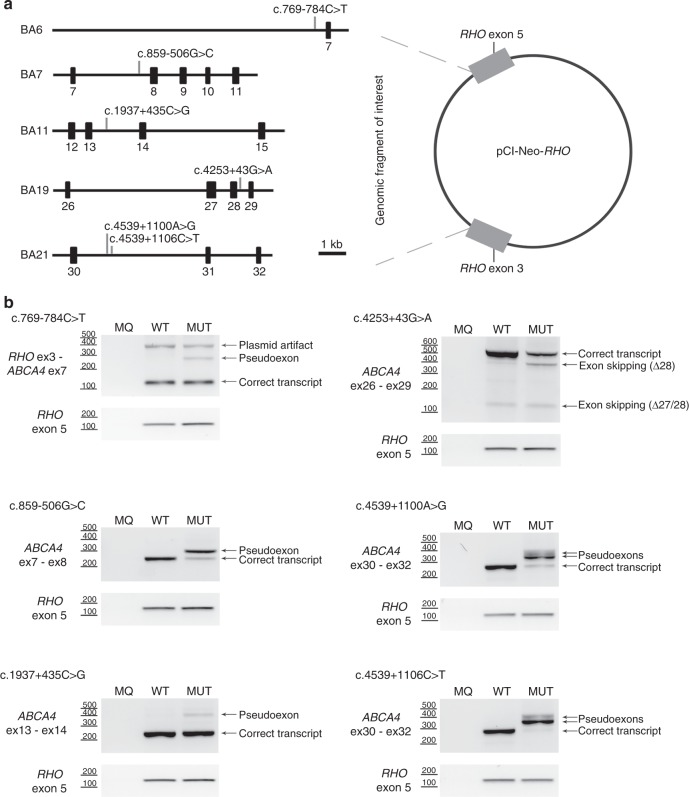
**Generation and assessment of splicing defects using midigenes.** **a** Schematic representation of the five wild-type midigenes used that were cloned between exons 3 and 5 of Rhodopsin in pCI-Neo-*RHO*. Positions of the variants present in six mutant midigenes are indicated. **b** Assessment of the splicing defects upon midigene transfection in HEK293T cells. Five pseudoexon (PE) inclusions and one exon skipping event were detected in the mutant (MUT) constructs compared to the wild-type (WT). MQ stands for the negative control of the polymerase chain reaction (PCR). Rhodopsin (*RHO*) amplification was used as a transfection and loading control.

To test the functional effect of the 11 selected noncoding variants, seven *ABCA4* wild-type midigene constructs previously described as BA4, BA6, BA7, BA9, BA11, BA19, and BA21^30^ and the newly designed BA30 (Table S5) were mutagenized. Upon RT-PCR and Sanger sequence validation, four variants had no effect (Figure S1). Variant c.858+526T>G did not show a PE insertion, but did yield four weak smaller fragments (Figure S1), the relevance of which is unknown in the absence of sequence results. This variant was found in *cis* with a complex allele containing a protein-truncating pathogenic variant (p.[Trp273*; Asn1868Ile]) in individual E-II:1, as determined by segregation analysis. The remaining six variants resulted in clear splice defects (Fig. 1, Figure S2) that allowed us to detect the second variant in 15/26 monoallelic probands, two deep-intronic variants in 2/3 cases without *ABCA4* variants, and a complex allele (c.[769-784C>T; 5603A>T]) in seven STGD1 cases carrying one deep-intronic variant. Considering that these variants or complex alleles are causal, segregation was as expected (Table 1). The remaining 12 unsolved STGD1 cases are listed in Table S6.

**Table 1:** Persons with Stargardt disease (STGD1) carrying two pathogenic *ABCA4* alleles

Patient_ID	Gender	Age at onset (years)	Segregation confirmed	Allele1 DNA	Allele1 protein	Allele 2 DNA	Allele 2 protein
A-I:2	F	38	Yes	c.1822T>A	p.(Phe608Ile)	c.[769-784C>T; 5603A>T]	p.[=, Leu257Aspfs*3; Asn1868Ile]^b^
B-I:2	F	10	Yes	c.768G>T	p.(Leu257Valfs*17)^c^	c.859-506G>C	p.[Phe287Thrfs*32, =]^b^
C-I:2	F	48	Yes	c.768G>T	p.(Leu257Valfs*17)^c^	c.[769-784C>T; 5603A>T]	p.[=, Leu257Aspfs*3; Asn1868Ile]^b^
D-III:1	M	45	Yes	c.4363T>C	p.(Cys1455Arg)	c.[4253+43G>A; 6006-609T>A]	p.[=, Ile1377Hisfs*3]^b^
E-II:1	F	7	Yes	c.[818G>A; 5603A>T]	p.[Trp273*; Asn1868Ile]	c.4539+1100A>G	p.[Arg1514Valfs*31, Arg1514Glyfs*3, =]^b^
F-II:2	F	20	Yes	c.1822T>A	p.(Phe608Ile)	c.[4253+43G>A; 6006-609T>A]	p.[=, Ile1377Hisfs*3]^b^
G-I:2	F	51	Yes	c.1822T>A	p.(Phe608Ile)	c.[4253+43G>A; 6006-609T>A]	p.[=, Ile1377Hisfs*3]^b^
H-I:2	F	53	Yes	c.[5461-10T>C; 5603A>T]	p.[Thr1821Valfs*13, Thr1821Aspfs*6; Asn1868Ile]	c.[4253+43G>A; 6006-609T>A]	p.[=, Ile1377Hisfs*3]^b^
I-II:3	F	44	Yes	c.4577C>T	p.(Thr1526Met)	c.[769-784C>T; 5603A>T]	p.[=, Leu257Aspfs*3; Asn1868Ile]^b^
J-II:3	M	61	Yes	c.768G>T	p.(Leu257Valfs*17)^c^	c.[4253+43G>A; 6006-609T>A]	p.[=, Ile1377Hisfs*3]^b^
K-II:1	M	62	Yes	c.6155del	p.(Asn2052Thrfs*9)	c.[769-784C>T; 5603A>T]	p.[=, Leu257Aspfs*; Asn1868Ile]^b^
L-II:1	M	61	Yes	c.[5461-10T>C; 5603A>T]	p.[Thr1821Valfs*13, Thr1821Aspfs*6; Asn1868Ile]	c.[769-784C>T; 5603A>T]	p.[=, Leu257Aspfs*; Asn1868Ile]^b^
M-II:1	M	18	Yes	c.4539+2001G>A	p.[=, Arg1514Leufs*36]^d^	c.[4253+43G>A; 5603A>T]	p.[=, Ile1377Hisfs*3; Asn1868Ile]^b^
N-II:3	M	31	Yes	c.4773+1G>A	p.(?)	c.[4253+43G>A; 6006-609T>A]	p.[=, Ile1377Hisfs*3]^b^
O-I:1	M	49	Yes	c.3113C>T	p.(Ala1038Val)	c.859-506G>C	p.[Phe287Thrfs*32, =]^b^
P-II:3	F	4	Yes	c.5196+1137G>A	p.[Met1733Glufs*78, =]^e^	c.859-506G>C	p.[Phe287Thrfs*32, =]^b^
Q-II:1	M	69	Yes	c.4539+1G>T	p.(?)	c.[769-784C>T; 5603A>T]	p.[=, Leu257Aspfs*3; Asn1868Ile]^b^
R-II:1	M	52	n.t.	c.4539+1G>T	p.(?)	c.[4253+43G>A; 6006-609T>A]	p.[=, Ile1377Hisfs*3]^b^
S-II:1	F	64	n.t.	c.768G>T	p.(Leu257Valfs*17)^c^	c.[769-784C>T; 5603A>T]	p.[=, Leu257Aspfs*3; Asn1868Ile]^b^
T-II:1	F	35	Yes	c.[2588G>C; 5603A>T]	p.[Gly863Ala, Gly863del; Asn1868Ile]	c.1937+435C>G	p.[=, Ser646Serfs*25]^b^
U-II:1	M	9	n.t.	c.768G>T	p.(Leu257Valfs*17)^c^	c.1937+435C>G	p.[=, Ser646Serfs*25]^b^
V-II:1	F	15	n.t.	c.[2588G>C; 5603A>T]	p.[Gly863Ala, Gly863del; Asn1868Ile]	c.4539+1100A>G	p.[Arg1514Valfs*31, Arg1514Glyfs*3, =]^b^
W-II:1	n.a.	11^a^	n.t.	c.[1622T>C; 3113C>T]	p.[Leu541Pro; Ala1038Val]	c.4539+1106C>T	p.[Arg1514Valfs*31, Arg1514Glyfs*3]^b^
X-II:1	n.a.	52^a^	n.t.	c.4469G>A	p.(Cys1490Tyr)	c.[4253+43G>A; 6006-609T>A]	p.[=, Ile1377Hisfs*3]^b^

n.t., not tested

^a^Unknown age of onset. Current age

^b^Predicted based on densitometry percentages in this study based on the method described in Sangermano et al.^30^

^c^Effect based on Sangermano et al.^30^

^d^Effect based on Albert et al.^22^

^e^Predicted based on Braun et al.^21^

The deep-intronic variant c.769-784C>T, present in 7/36 STGD1 cases, is located in intron 6, and was predicted to increase the strength of an intronic splice acceptor site at position g.94,549,775. In addition, a strong splice donor site was situated 161 nt downstream. RT-PCR analysis using wild-type and mutant minigene constructs revealed that, compared with the wild-type construct, the mutant c.769-784C>T construct showed an additional band of 296 bp, containing a 162-nt PE (Fig. 1b, Figure S2). Quantification of mutant mRNA in transfected HEK293T cells revealed that the PE fragment only accounted for ~8% of the total *ABCA4* pre-mRNA (Table S7), suggesting a mild effect at the RNA and thus protein level (r.[=, 768_769ins(162)]; p.[=, Leu257Aspfs*3]).

The deep-intronic variant c.859-506G>C was predicted to significantly strengthen a cryptic intronic splice acceptor site and is accompanied by a downstream splice donor site. This variant resulted in a 56-nt PE insertion (Fig. 1b, Figure S2), which accounted for 75.5% of total splice products (Table S7), indicating the highly deleterious effect of this variant at the RNA and (predicted) protein level (r.[858_859ins(56), =]; p.[Phe287Thrfs*32, =]).

Deep-intronic variants c.4539+1100A>G and c.4539+1106C>T were predicted to alter the same cryptic splice donor site located at position g.94,493,901. A very strong splice acceptor site was located at g.94,493,968. Besides the expected 68-nt PE fragment (PE30.1) due to the splice acceptor site at position c.4539+1033 and the splice donor site at position c.4539+1100, an additional 112-nt PE (PE30.2) carrying the same splice donor but another acceptor site located at g.94,494,012 was observed (Fig. 1b, Figure S2). Interestingly, the relative ratios of these two PEs significantly differed between the midigene constructs carrying c.4539+1100A>G or c.4539+1106C>T. PE30.1 accounted for 60.6% and 93.1% of the total transcript in c.4539+1100A>G or c.4539+1106C>T respectively, while PE30.2 was present in 20.1% and 3.8% of the complementary DNA (cDNA) products, respectively (Table S7). Because both c.4539+1100A>G and c.4539+1106C>T showed only a minor fraction of correctly spliced products (19.2% and 3.0%) and because PE30.1 and PE30.2 are both predicted to lead to a frameshift, these variants were both deemed to have a severe effect (r.[4539_4540ins(68), 4539_4540ins(112), =]; p.[Arg1514Glyfs*3, Arg1514Valfs*31, =]) (Table 1, Table S8).

### c.1937+435C>G results in PE generation through the disruption of putative splice silencers

The c.1937+435C>G variant did not alter the strength of a cryptic splice site. Instead, it was predicted to disrupt an ESE (SC35) motif, and, more importantly, to inactivate two splice silencer motifs and to lower the predicted strength of a third one (Figure S3). This variant was located within the genomic sequence of a low-abundance *ABCA4* transcript that contains intron 13 sequences.^21^ RT-PCR analysis revealed a 134-nt PE insertion between positions c.1937+396 and c.1937+529 in mutant BA11 compared with WT (Fig. 1b, Figure S2). Although the splice defect caused by this variant disrupted the reading frame (r.[=, 1937_1938ins(134)]; p.[=, Ser646Serfs*25]), the fraction of mutant transcript was low (8.6%) (Table S7).

### Variant c.4253+43G>A results in exon skipping through the disruption of putative splice silencers

The c.4253+43G>A variant was present in eight monoallelic STGD1 individuals and in one proband carrying a causal variant and p.Asn1868Ile in *trans*. It does not weaken the splice donor site of exon 28, nor does it affect any other cryptic splice sites in silico. It does affect the strength of several splice enhancer or silencer motifs (Figure S4). The c.4253+43G>A variant was found in *cis* with the previously reported c.6006-609T>A^11^ in eight of nine probands, but c.6006-609T>A showed no effect in an in vitro splice assay (Figure S2). The c.4253+43G>A mutant yielded bands of 495 and 370 bp, the first corresponding to the correctly spliced product, and the second to a product in which exon 28 was skipped (Fig. 1b, Figure S2). This 125-nt deletion is predicted to lead to a frameshift, and although both WT and mutant midigenes showed some skipping of exon 28, in the mutant construct this was significantly higher compared with WT (26.1% vs. 2.8%, Table S7). The fact that in all nine probands, a severe variant was found in *trans*, in combination with a late age of onset, suggests a mild effect of this variant (r.[=, 4129_4253del(125)]; p.[=, Ile1377Hisfs*3]). An overview of the allele frequencies and of the splice defects caused by all six variants can be found in Tables S8 and S9.

### smMIP-based sequence analysis of intronic *ABCA4* regions

Five of six intronic *ABCA4* variants were analyzed for their recurrence in 412 genetically unsolved STGD1 cases from France (*n* = 224) or Germany (*n* = 188), by employing smMIPs.^31^ Three variants were found in a heterozygous state in this cohort: c.769-784C>T (*n* = 4), c.4253+43G>A (*n* = 29), and c.4539+1106C>T (*n* = 1) (Table S8 and Table S9), whereas variants c.859-506G>C and c.4539+1100A>G were not detected.

### Antisense oligonucleotide-based exclusion of PEs

For five variants (c.769-784C>T, c.859-506G>C, c.1937+435C>G, c.4539+1100A>G, and c.4539+1106C>T), we designed three different AONs each that aim to result in PE exclusion. For c.4253+43G>A, only two AONs were designed, aiming to block splice silencer motifs to reinforce inclusion of exon 28. A summary of the sequences and characteristics of the AONs is provided in Table S2. The effect of the AONs at the RNA level was assessed by RT-PCR and subsequently semiquantified using capillary analysis (Figure S5, Table S7).

**Fig. 2:**
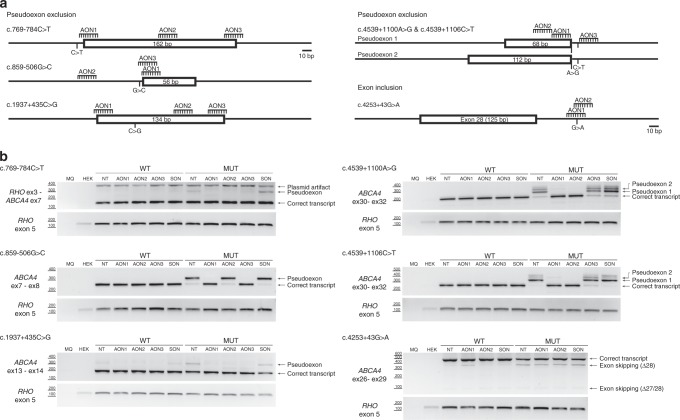
**Antisense oligonucleotide (AON) rescue using midigenes in HEK293T cells.** **a** Position of the AONs and sizes of the pseudoexons introduced by the variants. **b** Assessment of the splicing correction by reverse transcription polymerase chain reaction (RT-PCR) upon AON delivery. Wild-type (WT) midigenes and mutant (MUT) midigenes were cotransfected with different AONs and a SON, except the nontransfected cells lane (NT). For each variant introducing a pseudoexon, at least one AON was able to redirect splicing. In the case of exon 28 skipping, a 10% inclusion was achieved with AON2. MQ denotes the negative control of the PCR reaction and HEK for the untransfected HEK293T. Rhodopsin (*RHO)* amplification was used as a transfection and loading control.

For c.769-784C>T, AON2 completely restored correct splicing, whereas AON3 had a partial effect (Fig. 2b). When using AON1, also some splice redirection was observed; an additional faint band was present both in WT and mutant. Cloning and sequence analysis of this band revealed that the 5’ 68 nt of the PE were skipped, while the 3’ 94 nt remained. Given the position of AON1, this AON most likely blocks the splice acceptor site of the PE, triggering the use of a downstream splice acceptor site. For c.859-506G>C, two AONs (AON1 and AON3) corrected the splicing defect (Fig. 2b). For c.1937+435C>G, all three AONs restored normal splicing (Fig. 2b). In addition, we observed that the 134-nt PE could also naturally be included in the WT construct, as the band was also present in the nontreated and the SON-treated samples. Since the PE is also inserted when transfecting the WT construct, the variant apparently enhances PE recognition. Finally, for the two neighboring variants c.4539+1100A>G and c.4539+1106C>T, AON1 and AON2 correctly restored splicing, while AON3 did not show any rescue (Fig. 2b).

### AON-based PE exclusion using STGD1-derived fibroblast cells

**Fig. 3:**
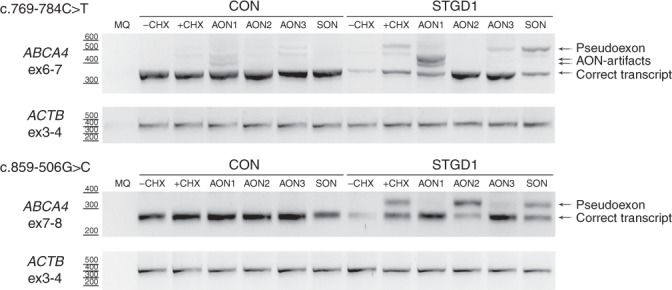
**Antisense oligonucleotide (AON) rescue in fibroblasts derived from Stargardt disease (STGD1)-affected individuals carrying c.769-784C>T or c.859-506G>C.** Fibroblast cells from a control (CON) and a STGD1 individual were transfected with three AONs and a SON. To detect the pseudoexon, cells were subjected to cycloheximide (+CHX) treatment. Only the nontransfected cells were not subjected to CHX treatment (-CHX). The MQ lane contains the negative control of the PCR reaction. Actin (*ACTB*) was used as a loading control.

To investigate whether the AON-based splice corrections observed in the midigene assays would also occur in patient-derived cells, we generated fibroblast cell lines from a proband carrying c.[769-784C>T; 5603A>T] and c.1822T>A (p.Phe608Ile) (patient A-I:2), and from a STGD1 proband carrying c.859-506G>C and a putative deletion on the other allele encompassing intron 7 (case DNA14-33085; not listed in Table 1). For both cases, the same PEs were detected as in the midigene assays (Fig. 3), yet a larger amount of PE-containing mRNA was detected in fibroblasts from A-I:2 grown under NMD-suppressing conditions (18.2%) compared with HEK293T-transfected cells (8.6%). Considering that this STGD1 individual carries a missense variant (p.Phe608Ile), the amount of PE-containing mRNA from the c.769-784C>T allele likely is much higher than 18%. The same AONs that restored splicing in the midigenes also showed rescue in fibroblasts (Fig. 3). For c.769-784C>T, AON2 was the most effective, correcting 100% of the transcripts, whereas AON3 also showed a strong rescue (Fig. 3, Figure S6, Table S10). AON1 also resulted in PE skipping but induced an alternative splicing event. Interestingly, the insertion of this partial PE was also detected in control fibroblasts. Sequence analysis revealed that the upper band of the artifact corresponded to the partial PE, while the lower was a heteroduplex consisting of the WT and partial PE-containing transcripts. *ABCA4* mRNA was also barely detected in the fibroblasts carrying c.859-506G>C when they were not subjected to CHX treatment (Fig. 3). After CHX incubation, a 56-nt PE was detected that, upon delivery of AON1 and AON3, was almost completely absent from the transcripts.

### AON-based exon inclusion

To correct the splice defect of c.4253+43G>A, AONs were designed to promote exon 28 inclusion. Two AONs were delivered to the midigene-transfected HEK293T cells. To assess whether the AON was inducing the inclusion of exon 28, the ratio between correct and Δexon 28 transcript was calculated with Fiji, or using the corrected peak area obtained in the capillary analysis (Figure S5). Using both methods, AON2 was shown to induce ~10% extra inclusion of exon 28 (Fig. 2), whereas for AON1, splice redirection was less evident (Table S7).

## DISCUSSION

To identify missing noncoding variants in STGD1 cases, we sequenced the *ABCA4* locus in 36 probands and identified one known and five novel intronic variants in 24 (67%) probands. Four novel deep-intronic variants (c.769-784C>T, c.859-506G>C, c.4539+1100A>G, c.4539+1106C>T) strengthen cryptic splice sites at noncanonical nucleotide positions and thereby result in PE insertions, contrary to most of the published deep-intronic splice site variants that create canonical splice sequences.^34,35^ Variant c.1937+435C>G disrupted a splicing silencer motif that is located within a low-expressed alternate exon, and resulted in a 134-nt PE formation.^21^ This PE contains a proper splice acceptor site but, similar to the alternate exon, no consensus splice donor site sequence. The c.4253+43G>A variant disrupted predicted splice silencers and created an ESE, and led to partial skipping of the upstream exon 28. Interestingly, we identified two probands (M-II:1 and P-II:3) that carried deep-intronic variants on both alleles.

All novel variants were predicted to result in protein truncation, but two variants, c.769-784C>T and c.4253+43G>A, found in seven and nine probands, respectively, only affect a small proportion of the mRNA. Testing of 412 other genetically unexplained STGD1 cases revealed 29 persons to carry c.4253+43G>A and four cases to carry c.769-784C>T. AONs were able to (partially) correct the observed splice defects for all variants in HEK293T cells and, for two variants, in STGD1-derived fibroblasts.

Fourteen first alleles in the 24 probands carry protein-truncating variants and are thus assumed to have a severe effect (Table 1). For other missense or complex alleles, the severity is less clear. Based on previous studies,^15,36,37^ our mRNA analysis of the deep-intronic variants, and the ages at onset of the STGD1 probands described here, we can hypothesize on the severity of the intronic variants. The c.859-506G>C, c.4539+1100A>G, and c.4539+1106C>T variants all show PE insertion in the majority of *ABCA4* transcripts and can thus be considered severe variants. This correlates very well with an age at onset ≤10 years when found in *trans* with a severe variant and between 15 and 49 years when in *trans* with a mild variant (Table 1).

For c.1937+435C>G, the picture is more complicated. The splice defect was mild, and the two probands carrying this variant carry either a severe variant p.(Leu257Valfs*17) (U-II:1) or a mild allele p.[Gly863Ala, Gly863del; Asn1868Ile] (T-II:1) in *trans*. The ages of onset were 9 and 35 years, respectively, which argues for a severe nature of c.1937+435C>G in U-II:1 and a moderate-to-severe nature in T-II:1. This is discordant with the observed partial PE insertion in HEK293T cells. A similar observation was recently made while studying the effect of c.4539+2001G>A and c.4539+2028C>T.^21^ In STGD1-derived fibroblasts, mRNA analysis did not show any PE insertion. In STGD1-derived photoreceptor precursor cells however, we observed up to 15% (c.4539+2028C>T) and 25% (c.4539+2001G>A) of PE-containing mRNA.^22^ Based on their heterozygous presence in the corresponding STGD1 cases and their moderate-to-severe (c.4539+2028C>T) or severe (c.4539+2001G>A) nature, about two times these percentages were expected. We hypothesize that the photoreceptor precursor cells had not yet reached a differentiation stage that closely mimics the human retina situation. It is also of interest that, while c.1937+435C>G is predicted to disrupt ESEs, c.4539+2001G>A and c.4539+2028C>T are predicted to *activate* ESEs. Possibly, the action of these splicing motifs requires specific factors that are exclusively present in mature photoreceptors, while being absent in HEK293T cell and explain the small effect for c.1937+435C>G. Patient-derived photoreceptor-like cells may reveal a more complete splice defect.

Recently, it was determined that c.2588G>C (p.[Gly863Ala, Gly863del]) only acts as a penetrant pathogenic variant when present in *cis* with c.5603A>T (p.Asn1868Ile).^15^ The common p.(Asn1868Ile) variant was found as a second *ABCA4* allele in up to 50% of monoallelic STGD1 cases and is strongly associated with late-onset STGD1.^15^ Upon Haloplex-based *ABCA4* gene sequencing, we found p.Asn1868Ile as a single variant in *trans* with other (potentially) causal variants in 25/65 (38%) of probands, who had an average age at onset of 42 years. In addition, we identified four biallelic but unaffected persons and calculated that this variant, when present in *trans* with a loss-of-function *ABCA4* variant, shows a penetrance <5%.^16^ Based on the relatively high frequency of c.769-784C>T in non-Finnish European controls (AF 0.00426 in gnomAD [http://gnomad.broadinstitute.org/]), its partial effect on splicing, and the very late age of onset observed in compound heterozygotes carrying c.[769-784C>T; 5603A>T] (61.0 years; range: 38–69 years), we consider that c.769-784C>T without c.5603A>T in *cis* may not be causative. We therefore investigated the MAFs of the c.769-784C>T and c.5603A>T variants (single and in combination) in 250 control Dutch families (GoNL: http://www.nlgenome.nl/).^38^ The single c.769-784C>T allele was found more often (6/998 alleles) than the c.[769-784C>T; 5603A>T] allele (2/998 alleles). The allele frequency of c.[769-784C>T; 5603A>T] in our STGD1 patient cohort (*n* = 250), 0.014, is significantly higher than in our general population (Chi-square test: *p* = 0.023; Bonferroni corrected). In two previous studies, the mean ages at onset for STGD1 patients carrying c.5603A>T as a single second allele in *trans* with severe *ABCA4* variants were 36 years^15^ and 42 years.^16^ The mean age of onset for STGD1 patients carrying c.[769-784C>T; 5603A>T] is higher than the age of onset of patients carrying the single c.5603A>T variant (Mann–Whitney U; *p*<0.05). Given its relatively small effect on mRNA splicing (18.2% PE inclusion in mRNA from patient carrying this variant in a heterozygous manner) and its invariable *cis*-configuration with c.5603A>T, we cannot unequivocally assign pathogenicity to c.769-784C>T. Possibly, patient-derived photoreceptor progenitor cells may shed further light on this matter.

Interestingly, c.4253+43G>A also shows a relatively high non-Finnish European AF (0.00598 in gnomAD). Although c.4253+43G>A, with one exception (M-II:1), was found in *cis* with c.6006-609T>A, we did not observe a splice defect for the latter variant in vitro. This could be due to the lack of retina-specific splice factors in the tested cells or due to another missed deep-intronic variant that acts in concert with c.4253+43G>A. In the one proband with c.4253+43G>A who lacked c.6006-609T>A, the c.5603A>T (p.Asn1868Ile) variant was found in *cis*. The age of onset of the cases carrying c.4253+43G>A ranged from 18 to 61 years (average 41 years). Interestingly, the earliest age at onset (18 years) was observed in the proband M-II:1 who carried c.[4253+43G>A; 5603A>T].

The use of AONs to exclude PEs has been used for several IRD-associated genes.^24–29^ However, in this study, several technical challenges were observed. For the c.859-506G>C variant, the design of the AONs was limited to very small parts of the PE due to the repetitive nature of parts of the PE. In case of the c.4539+1100A>G and c.4539+1106C>T variants, we aimed to find effective AONs that would target the PE for both variants. In addition, using midigenes for c.769-784C>T, we observed variability between replicates, probably due to differences in the transfection efficiencies or passage of the cells. Nevertheless, for each variant, at least one effective AON was discovered.^17^

Despite the significant yield of novel *ABCA4* variants in this study, a second *ABCA4* variant was not detected in 11 monoallelic probands and there was one case with no *ABCA4* variants, which could be due to (1) the presence of heterozygous copy-number variations (CNVs), as only three were tested using CNV analysis; (2) missed noncoding variants residing in noncovered sequences; (3) too-stringent selection criteria when using splicing algorithms; (4) incomplete sensitivity of the midigene in vitro splice assay; and (5) genocopies in view of the high AF of *ABCA4* variants (5–10%) in the general population.^18,36,39^ Additional studies will be needed to further unravel the missing heritability of STGD1.

In conclusion, we identified deep-intronic variants in 24/36 (67%) of STGD1 cases with no *ABCA4* variants, one variant, or one causal variant in *trans* with c.5603A>T. Two alleles (c.[769-784C>T; 5603A>T] and c.4253+43G>A) are frequent in our STGD1 cohort and appear to be associated with late-onset STGD1. Due to its small effect on *ABCA4* mRNA in HEK293T cells and patient-derived fibroblasts, and its invariable presence on the same allele as c.5603A>T, we could not establish causality for c.769-784C>T. Interestingly, all the observed splice defects could be rescued with at least one of the tested AONs, which provides a basis for the development of new therapeutic strategies for individuals with STGD1 carrying these variants.

## Supplementary information

Supplementary information
