# Supplementary material for: Deep-intronic ABCA4 variants explain missing heritability in Stargardt disease and allow correction of splice defects by antisense oligonucleotides
Source: Genet Med. 2019 Jan 15;21(8):1751–60. doi: 10.1038/s41436-018-0414-9 (PMC6752325; doi:10.1038/s41436-018-0414-9)
Supplement: Supplementary file 1 — Supplementary information [file 41436_2018_414_MOESM1_ESM.pdf]

EXTENDED MATERIAL AND METHODS:

Samples

Twenty-five mono-allelic STGD1 individuals, eight cases carrying one causal *ABCA4* variant and p.Asn1868Ile in *trans*, and three probands with no *ABCA4* variant were genotyped. Thirty-two probands were ascertained from seven different medical centers in the Netherlands and Germany while four were ascertained in the United Kingdom, as part of the UK 10K sequencing project (https://www.uk10k.org/). Patients had been clinically diagnosed with Stargardt disease (STGD1) or cone-rod dystrophy (CRD) by an experienced ophthalmologist in the field of ophthalmogenetics. Each person manifested at least one of the following features of STGD1: yellow-white pisciform flecks in the retinal pigment epithelium of the posterior pole which were hyperautofluorescent on fundus autofluorescence imaging; and/or progressive atrophy of the macular retinal pigment epithelium. Age at onset for 22 STGD1 individuals and the current age of two STGD1 probands with intronic variants are given in Table 1. The age at onset for the remaining 12 STGD1 individuals is given in Supplementary Table S6. Age of onset was defined as the age at which the initial symptoms were noted by the person. For 17 unrelated Dutch probands, genomic DNA of 45 family members was obtained using Oragene DNA saliva kits (DNA Genotek Inc. Ottawa, Canada) as described by the manufacturer, to confirm segregation of non-coding variants.

A cohort of 412 French and German STGD1 individuals with one or no *ABCA4* variant was genotyped by using smMIPs to assess the recurrence of causal non-coding variants described in this study.

Written informed consent prior to participation in the study was obtained which adhered to the Declaration of Helsinki. This study was approved by the Medical Ethical Committee 2010-359 (Protocol nr. 2009-32; NL nr. 34152.078.10) and the Commissie Mensgebonden Onderzoek Arnhem-Nijmegen (Dossier no. 2015-1543; dossier code sRP4h).

***ABCA4* sequence analysis and selection of candidate splice variants**

For 22 mono-allelic cases, eight cases with one causal variant and c.5603A>T (p.Asn1868Ile) in *trans*, and two probands with no *ABCA4* variants, the entire 128-kb *ABCA4* gene (Chr1: 94,458,393 - 94,586,688) and approximately 117 kb proximal and 120 kb distal (total region: Chr1: 94,337,885 – 94,703,604) were enriched by using a custom Haloplex Target Enrichment kit (Agilent, Santa Clara, CA) followed by NGS. Probes to target the 365-kb region were designed with SureDesign (Agilent) and they covered >98% of the targeted regions; gaps in coverage corresponded with repeat-rich regions. Subsequently, samples were sequenced by using Illumina sequencing technology (MiSeq, Illumina, San Diego, CA, USA) to obtain 2x250 bp reads. Data analysis was performed with CLC Bio Software (CLC Bio Genomics Workbench, Qiagen, Hilden, Germany). Reads were aligned against a reference genome (GRCh37/hg 19 assembly) and variants were identified and annotated by a custom-designed workflow. Variants were only considered for further filtering if they were present in at least one forward and one reverse read, in a minimum of 20% of all reads and with a minimum coverage of five reads. The variants were further annotated by Alamut Batch v2.7 (Interactive Biosoftware). Prior to locus sequencing, the 32 STGD1 individuals were sequenced for variants in the coding and flanking splice site regions using Sanger sequencing and 20/32 cases also were analyzed for the presence of heterozygous CNVs using Multiplex Ligation Probe Amplification (MLPA). Probands carrying the p.Asn1868Ile common variant as a non-complex second allele (as based on segregation analysis) were excluded from this group earlier and will be described elsewhere.

Three mono-allelic cases (GC20009, GC17027, GC21017) and one proband with no *ABCA4* variants (GC20637) underwent genome sequencing as part of the National Institute for Health Research (NIHR) BioResource- Rare Diseases study[1] using the Illumina TruSeq DNA PCR-Free Sample preparation kit (Illumina, Inc.) and sequenced using an Illumina HiSeq 2500, generating minimum coverage of 15× for approximately 95% of the genome. Reads were aligned to the Genome Reference Consortium human genome build 37 (GRCh37) using Isaac Genome Alignment Software (version 01.14; Illumina, Inc.). Single nucleotide variants and small indels were identified using Isaac

Variant Caller (version 2.0.17). Structural variants were identified using two independent algorithms: Isaac Copy Number Variant Caller (Canvas, Illumina), and Isaac Structural Variant Caller (Manta, Illumina) as previously described.[1] In the first instance, coding variants with an AF <0.01 in control datasets including the NIHR BioResource Rare Diseases cohort and the Exome Aggregation Consortium (ExAC) database and passing standard quality filters were prioritized. A list of previously reported pathogenic mutations in relevant genes normally excluded by these filters were investigated (e.g., higher AF *ABCA4* variants: c.2588G>C, c.5882G>A, c.5461-10T>C. An in-house curated database of known and candidate IRD-associated genes was used to prioritize candidate biallelic variants in simplex and recessive families.

In all 36 samples, after exclusion of known first alleles, candidate variants were selected according to two inclusion criteria: i) presence in the *ABCA4* gene region (Chr1: 94,458,393 - 94,586,688), and ii) a MAF less than or equal to 0.01 in Genome of the Netherlands (GoNL; http://www.nlgenome.nl)[2], UK 10K project (https://www.uk10k.org/) and non-Finnish European gnomAD (http://gnomad.broadinstitute.org/) population frequency databases.

In silico splice site prediction for all variants was performed by using five algorithms (SpliceSiteFinder-like, MaxEntScan, NNSPLICE, GeneSplicer, Human Splicing Finder)[3-7] via Alamut Visual software version 2.7, by comparing splicing scores for WT and variant nucleotides. Inclusion criteria for splicing assays were: 1) the presence of a variant splice score >75% of the scoring range for at least two prediction programs, 2) a difference between wild-type and variant splice score >2% of the scoring range in at least two algorithms identified in 1). A few variants did not meet these criteria but were found enriched in our STGD1 cases or were located in alternate exons previously described [8] and therefore were included in the splice assays.

Cell lines and culture conditions

Human Embryonic Kidney 293 cells (HEK293T) were purchased from ECACC (Catalogue no. 12022001; Salisbury, UK), which were tested for genomic integrity by STR PCR. HEK293T cells were cultured in

Dulbecco's Modified Eagle Medium (DMEM), supplemented with 10% fetal calf serum (FCS), 1% penicillin-streptomycin (P/S) and 1% sodium pyruvate (NaPyr) at 37°C and 5% CO_2. Cells were split twice a week in a dilution 1:10. Fibroblast cell lines were generated in house from skin biopsies and cultured in DMEM supplemented with 20% FCS, 1% P/S and 1% NaPyr at 37°C and 5% CO_2. Cells were split twice a week in a dilution 1:3. For all cell lines Mycoplasma contamination tests were negative and were conducted every 2 months.

Midigene-based splice assay

The effect of 11 non-coding was assessed by midigene-based splicing assays employing seven wild-type BA clones previously described[9] and the newly designed BA30. All constructs contained *ABCA4* genomic sequences from the bacterial artificial chromosome clone CH17-325O16 (insert g.94,434,639-94,670,492) and they served as templates to generate mutant constructs by site-directed mutagenesis, which subsequently were validated by Sanger sequencing (Table S11 and S12). WT and mutant constructs were transfected in HEK293T cells and the extracted total RNA was subjected to reverse transcription (RT)-PCR (Table S1) as described previously.[9]

Single molecule Molecular Inversion Probe-based sequence analysis of intronic *ABCA4* regions

To test the occurrence of five intronic *ABCA4* variants (c.769-784C>T, c.859-506G>C, c.4253+43G>A, c.4539+1100A>G, c.4539+1106C>T) in 412 genetically unsolved STGD1 cases from Lille, France (n = 224) and Regensburg, Germany (n = 188), we designed smMIPs based on a previously developed cost-effective sequencing protocol (Table S13).[10] STGD1 samples from a French cohort previously were prescreened using a mutation scanning technique, dHPLC or NGS (i.e., sequence analysis of inherited retinal disease-associated variants, including *ABCA4*). The STGD1 cases from a German cohort were previously sequenced using a custom-designed GeneChip CustomSeq Resequencing Array (RetChip) (Affymetrix, Santa Clara, CA, USA) or NGS on an ION Torrent semiconductor personal

sequencing machine (Life Technologies, Darmstadt, Germany) upon multiplex-PCR amplification of the fragments with the STARGARDT MASTR kit (Multiplicom, Niel, Belgium).[11]

The genomic positions of the intronic variants were obtained from the UCSC genome browser (hg19; https://genome.ucsc.edu), and were used to design smMIPs using an in-house pipeline. For each intronic *ABCA4* variant, we designed two (overlapping) smMIPs, one on the plus strand and one on the minus strand. Each smMIP contains 78 nt and targets about 110 bp of genomic sequences based on the position of two annealing arms. The two annealing arms (denoted extension and ligation probes, together 40 nt) are connected using a common linker sequence of 38 nt which includes a 8-nt random tag that serves as the 'single molecule' identifier. The 8-nt random tags enable the recognition of individual capture events and thereby point to random sequencing errors. The probes were designed for each DNA strand separately to ensure coverage from the plus and minus strands. The 78-mer smMIPs were synthesized by Integrated DNA Technologies (IDT, Leuven, Belgium). All probes were pooled in equal amount (i.e., 1 µM), and thereafter used to prepare the sequencing libraries according to a protocol described elsewhere.[12]

We sequenced 412 STGD1 cases by using bar-coded PCR primers in two series. Bar-coded libraries were pooled, and after purification were sequenced using Illumina NexSeq500 using two Mid output kits (maximum of 130 million reads). The paired end sequencing reads were combined into 152-nt forward-reverse-reads (fr-reads), and were mapped to the reference genome. Next, the sm tags were exploited to group fr-reads. The resultant fr-read groups were used to make a highly accurate consensus read referred to as single molecule consensus reads (smc-reads), which were used for variant calling. The variant calling and annotation was performed using the in-house pipeline. The smc-read average ranged from 14 to 1587, with an average coverage of 489x.

Antisense Oligonucleotides

For each deep-intronic variant causing a PE inclusion three AONs were designed (Table S2). The design of the therapeutic molecules was performed as described elsewhere.[13,14] Briefly, for each

variant, the sequence of each PE plus the flanking regions (50 bp) were selected for further analyses. First the RNA structure and the exon splicing enhancers (ESEs) were predicted. Subsequently, AONs were designed taking several parameters into account (free energy, Tm, %GC, binding region, etc). For the c.4253+43G>A variant, two AONs were designed to bind directly on the mutant sequence. In addition, a sense oligonucleotide (SON) was synthesized. All oligonucleotides had a phosphorothioate backbone with a 2-*O*-methyl sugar modification (2OMe/PS) and were synthesized by Eurogentec.

All oligonucleotides (AONs and SON) were resuspended in PBS at a concentration of 100 µM (stock solution). The final concentration used in all the experiments was 0.5 µM.

***In vitro* rescue studies in HEK293T cells using midigenes and AONs**

Around 400,000 HEK293T cells were seeded in each well of a 6-well plate. The next day, each well was transfected with 1.5 µg of either the wild-type or the mutant construct. In total, eleven wells were transfected with one of the midigene constructs, while one well was left untransfected (HEK293T control). Of note, variants c.4539+1100A>G and c.4539+1106C>T result in the insertion of the same PEs and therefore were inserted in the same wild-type construct and were treated with the same AONs. Twenty-four hours post-transfection, each well was trypsinised and subdivided in 5 wells of a 24-well plate. Once cells were attached, the cells of each well were transfected with the respective AON, the SON, or were left untransfected (NT). Transfections were performed using FuGENE® HD Transfection Reagent (Promega) using a ratio 3:1 (3 µl of reagent for each µg of DNA) for midigenes and 1:10 for AONs (1 µl of reagent for each 10 µl of AON). Forty-eight hours post-AON delivery, cells were harvested and RNA isolation was performed using NucleoSpin® RNA kit (Macherey-Nagel). Subsequently, 1 µg of RNA was used for cDNA synthesis using iSCRIPT cDNA synthesis kit (Bio-Rad). cDNA was diluted to 20 ng/µl by adding 30 µl of MQ and 50 ng (2.5 µl) was used for the PCR analysis. All PCR reactions were prepared at a final volume of 25 µl. The conditions for all PCR reactions were as follows: 2 min at 94°C, followed by 35 cycles of 30 s at 94°C, 30 s at 58°C

and 2 min at 72°C, a final elongation step of 5 min at 72°C was performed. Subsequently, 10 µl of each reaction were loaded onto a 2%-agarose gel. Rhodopsin amplification was used as transfection and loading control. All experiments were performed in two independent replicates. Primer sequences can be found in the Table S1.

Rescue studies using antisense oligonucleotides in fibroblasts

Approximately 500,000 fibroblast cells were seeded in each well of a 6-well plate (6 wells per cell line). The next day, cells were transfected with either 0.5 µM of one of the AONs or SON, or transfected with empty liposomes (NT). Forty-four hours post-transfection medium was removed and medium containing cycloheximide (CHX) was added in order to block non-sense mediated decay (NMD). Four hours later cells were harvested and subjected to RNA isolation as previously described. For cDNA synthesis, 1 µg of RNA was used using Invitrogen™ SuperScript™ IV VILO™ Master Mix (Thermo Fisher Scientific). cDNA was diluted to 20 ng/µl by adding 30 µl of MQ. For each PCR reaction, 80 ng (4 µl) of cDNA were used, except for actin (50 ng). All PCR reactions were prepared at a final volume of 25 µl. The conditions for all *ABCA4* PCR reactions were as follows: 2 min at 94°C, followed by 40 cycles of 30 s at 94°C, 30 s at 58°C and 2 min at 72°C, a final elongation step of 5 min at 72°C was performed. For actin, the conditions were as follows: 2 min at 94°C, followed by 35 cycles of 30 s at 94°C, 30 s at 58°C and 30 s at 72°C, a final elongation step of 2 min at 72°C. Subsequently, 20 µl of each *ABCA4* reaction were loaded onto a 2%-agarose gel. Ten microliter of the amplified actin product was used as loading control. All experiments were performed in two independent replicates. Primer sequences are indicated in Table S1.

Capillary analysis

In order to quantify the ratios between correct and aberrant transcripts we loaded the RT-PCR samples onto a Fragment Analyzer Auto Capillary Electrophoresis System (Advanced Analytical Technologies, Inc.). Midigene-derived RT-PCR products were diluted 1:10 whereas fibroblast-derived

RT-PCRs were diluted 1:4. In both cases 2 µl was used for capillary analysis. Analysis of the peaks was performed using the corrected peak area parameter and only the peaks corresponding to the expected bands were taken into account.

References

1. Carss KJ, Arno G, Erwood M, et al. Comprehensive Rare Variant Analysis via Whole-Genome Sequencing to Determine the Molecular Pathology of Inherited Retinal Disease. *American journal of human genetics.* 2017;100(1):75-90.
2. Genome of the Netherlands C. Whole-genome sequence variation, population structure and demographic history of the Dutch population. *Nature genetics.* 2014;46(8):818-825.
3. Desmet FO, Hamroun D, Lalande M, Collod-Beroud G, Claustres M, Beroud C. Human Splicing Finder: an online bioinformatics tool to predict splicing signals. *Nucleic acids research.* 2009;37(9):e67.
4. Pertea M, Lin X, Salzberg SL. GeneSplicer: a new computational method for splice site prediction. *Nucleic acids research.* 2001;29(5):1185-1190.
5. Reese MG, Eeckman FH, Kulp D, Haussler D. Improved splice site detection in Genie. *J Comput Biol.* 1997;4(3):311-323.
6. Shapiro MB, Senapathy P. RNA splice junctions of different classes of eukaryotes: sequence statistics and functional implications in gene expression. *Nucleic acids research.* 1987;15(17):7155-7174.
7. Yeo G, Burge CB. Maximum entropy modeling of short sequence motifs with applications to RNA splicing signals. *J Comput Biol.* 2004;11(2-3):377-394.
8. Braun TA, Mullins RF, Wagner AH, et al. Non-exomic and synonymous variants in ABCA4 are an important cause of Stargardt disease. *Human molecular genetics.* 2013;22(25):5136-5145.
9. Sangermano R, Khan M, Cornelis SS, et al. ABCA4 midigenes reveal the full splice spectrum of all reported noncanonical splice site variants in Stargardt disease. *Genome Res.* 2018;28(1):100-110.
10. Hiatt JB, Pritchard CC, Salipante SJ, O'Roak BJ, Shendure J. Single molecule molecular inversion probes for targeted, high-accuracy detection of low-frequency variation. *Genome Res.* 2013;23(5):843-854.
11. Schulz HL, Grassmann F, Kellner U, et al. Mutation Spectrum of the ABCA4 Gene in 335 Stargardt Disease Patients From a Multicenter German Cohort-Impact of Selected Deep Intronic Variants and Common SNPs. *Investigative ophthalmology & visual science.* 2017;58(1):394-403.
12. O'Roak BJ, Vives L, Fu W, et al. Multiplex targeted sequencing identifies recurrently mutated genes in autism spectrum disorders. *Science.* 2012;338(6114):1619-1622.
13. Aartsma-Rus A. Overview on AON design. *Methods in molecular biology.* 2012;867:117-129.
14. Garanto A, Collin RWJ. Design and In Vitro Use of Antisense Oligonucleotides to Correct Pre-mRNA Splicing Defects in Inherited Retinal Dystrophies. *Methods in molecular biology.* 2018;1715:61-78.

Fig. S1

Fig. S1. Absence of splice defects for five deep-intronic variants in *ABCA4*. Five deep-intronic variants were inserted in four BA constructs. RT-PCR was performed by using *ABCA4* exonic primers for BA7 and BA30, whereas for variants in BA4 and BA9 we used both rhodopsin (*RHO)* primers.

BA4

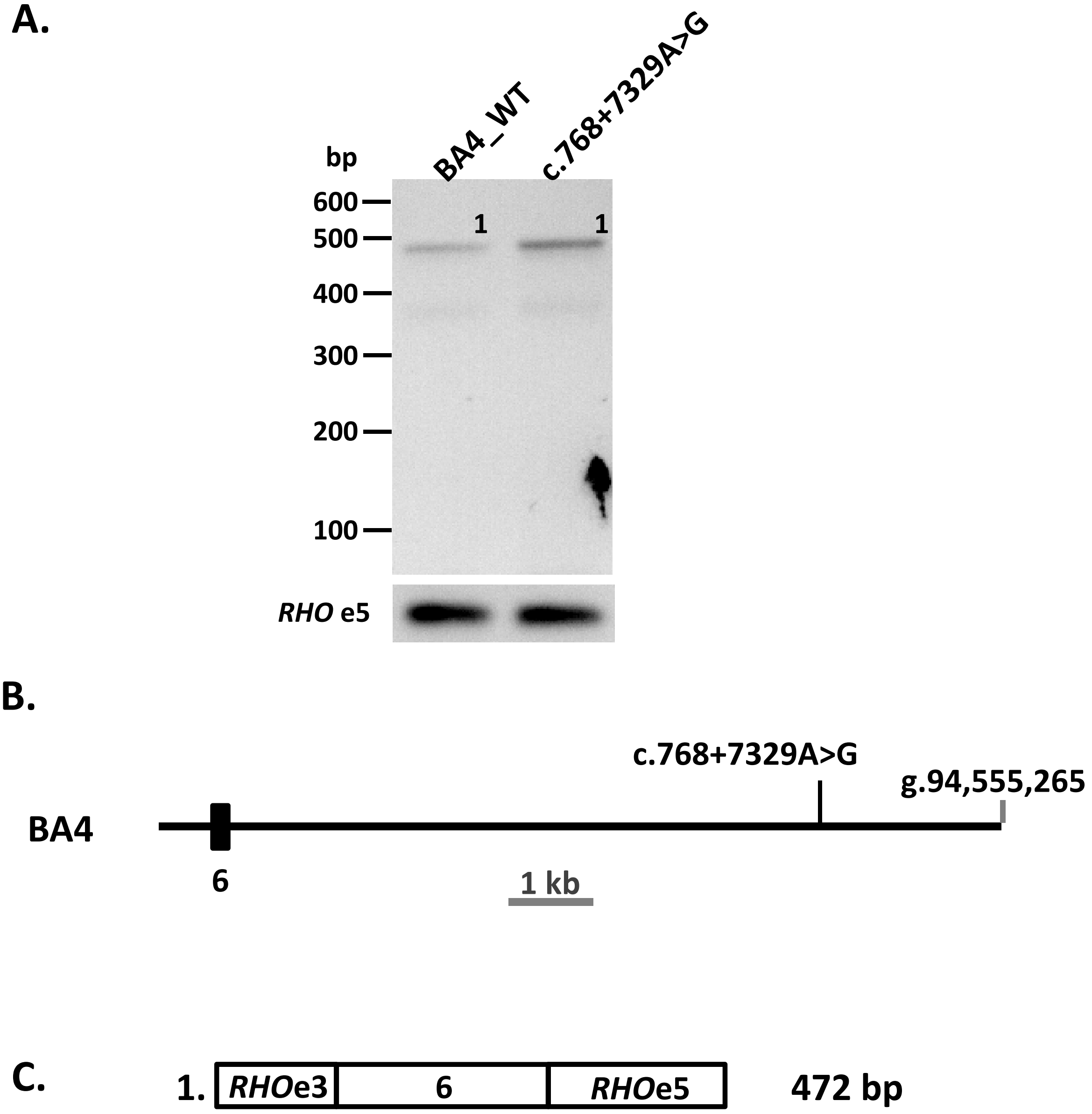
A.
bp
BA4_WT
c.768+7329A>G
600
500
400
300
200
100
1
1
RHO e5
B.
c.768+7329A>G
g.94,555,265
BA4
6
1 kb
C.
1.
RHOe3
6
RHOe5
472 bp

BA7

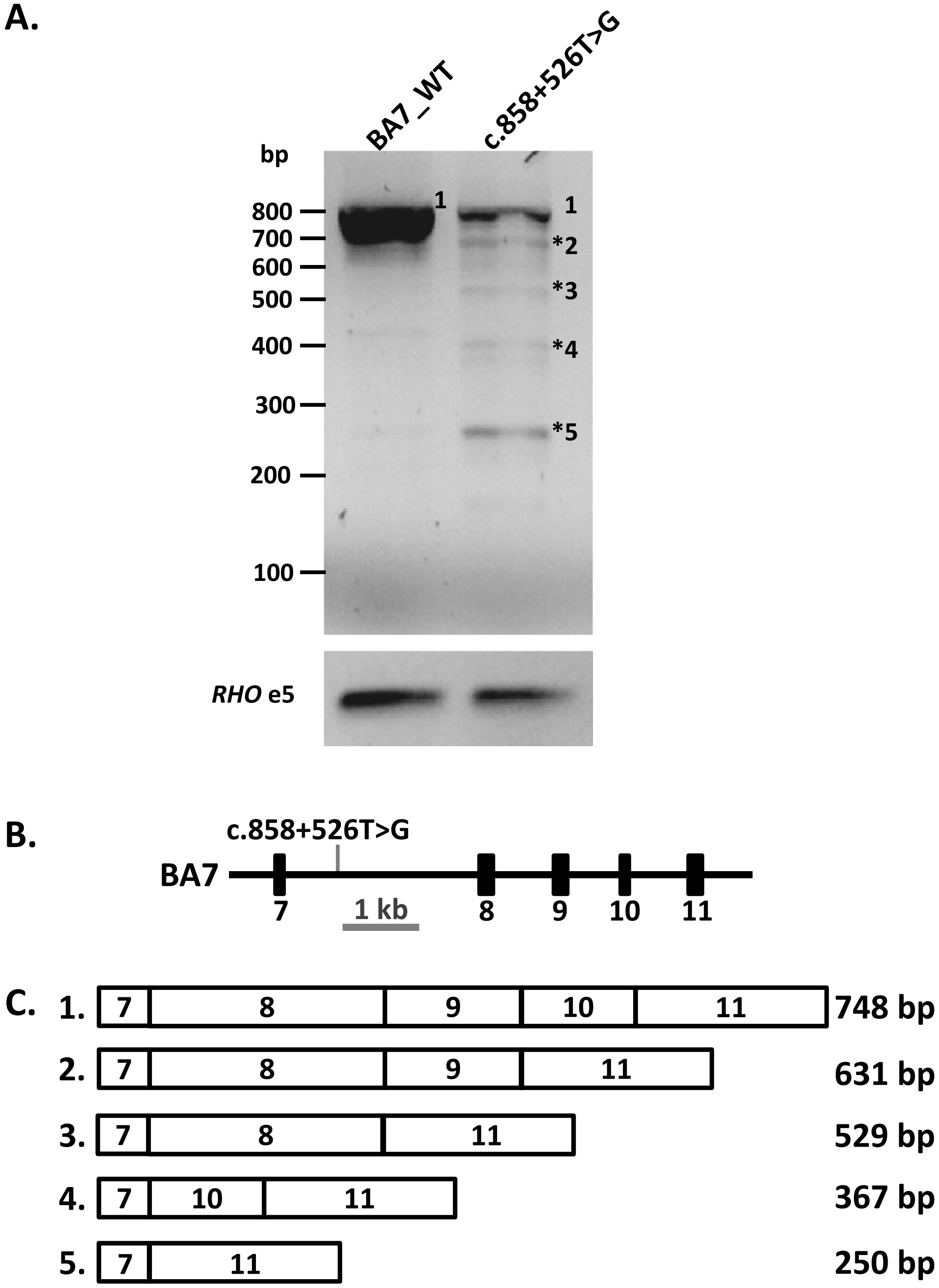

*No sequence information available

BA9

A.

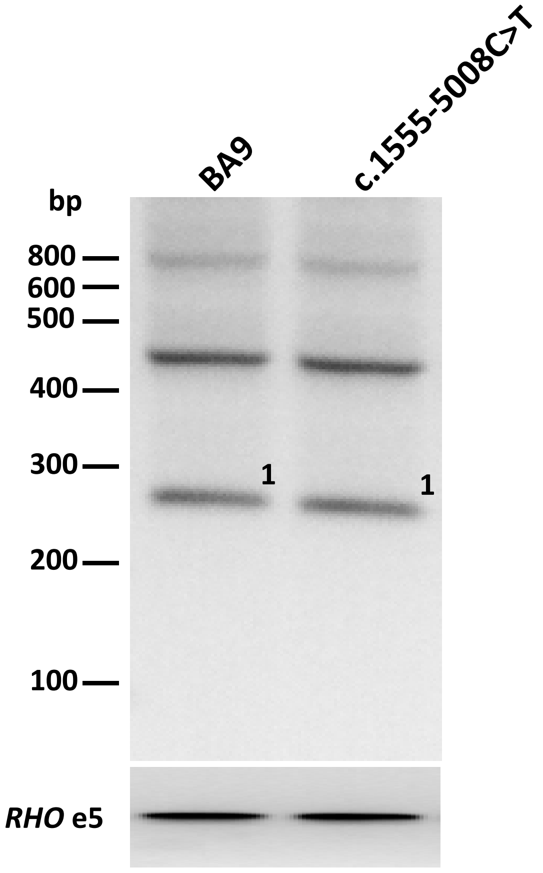
BA9
c.1555-5008C>T
bp
800
600
500
400
300
200
100
1
1
RHO e5

B.

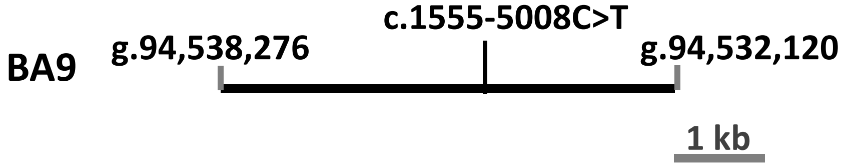
BA9
g.94,538,276
c.1555-5008C>T
g.94,532,120
1 kb

C.

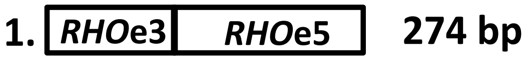
1.
*RHO*e3
*RHO*e5
274 bp

BA30

A.

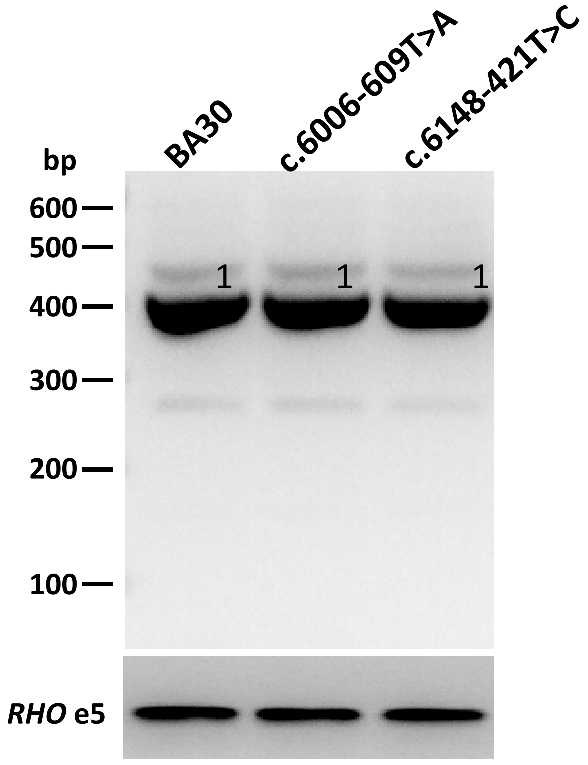
BA30
c.6006-609T>A
c.6148-421T>C
bp
600
500
400
300
200
100
1
1
1
RHO e5

B.

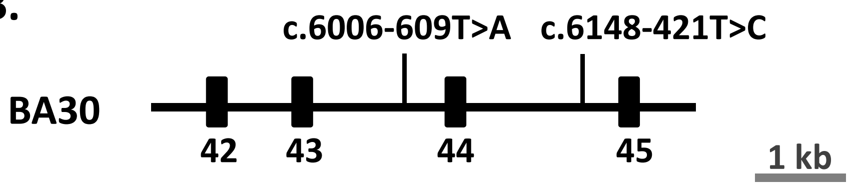
c.6006-609T>A
c.6148-421T>C
BA30
42
43
44
45
1 kb

C.

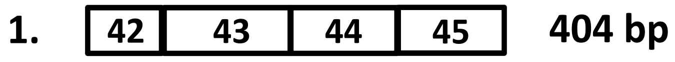
1.
42
43
44
45
404 bp

Fig. S2

Fig. S2. Sequence analysis of RT-PCR products from *in vitro* splice assays. Sanger sequenced electropherograms for six causal non-coding variants and wild-types (WT) are given. All the electropherograms are named according to the fragments in Fig. 1. Electropherograms obtained after gel purification are indicated by letter “G” encircled in green while letter “P” encircled in blue indicates the results of pGEM cloned bands.

BA6

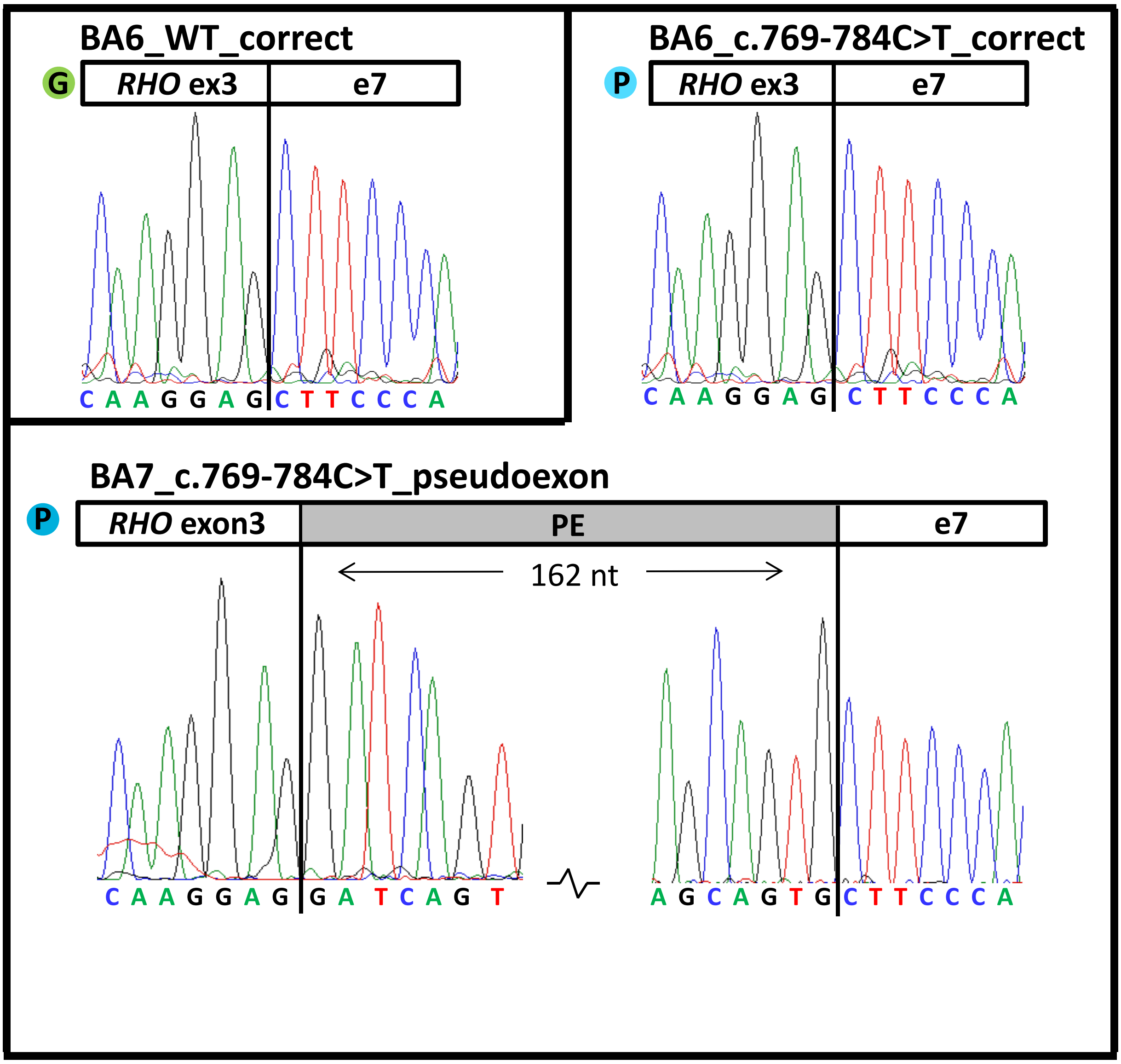

BA6_WT_correct
G
RHO ex3
e7
C A A G G A G C T T C C C A
BA6_c.769-784C>T_correct
P
RHO ex3
e7
C A A G G A G C T T C C C A
BA7_c.769-784C>T_pseudoexon
P
RHO exon3
PE
e7
162 nt
C A A G G A G G A T C A G T
A G C A G T G C T T C C C A

BA7

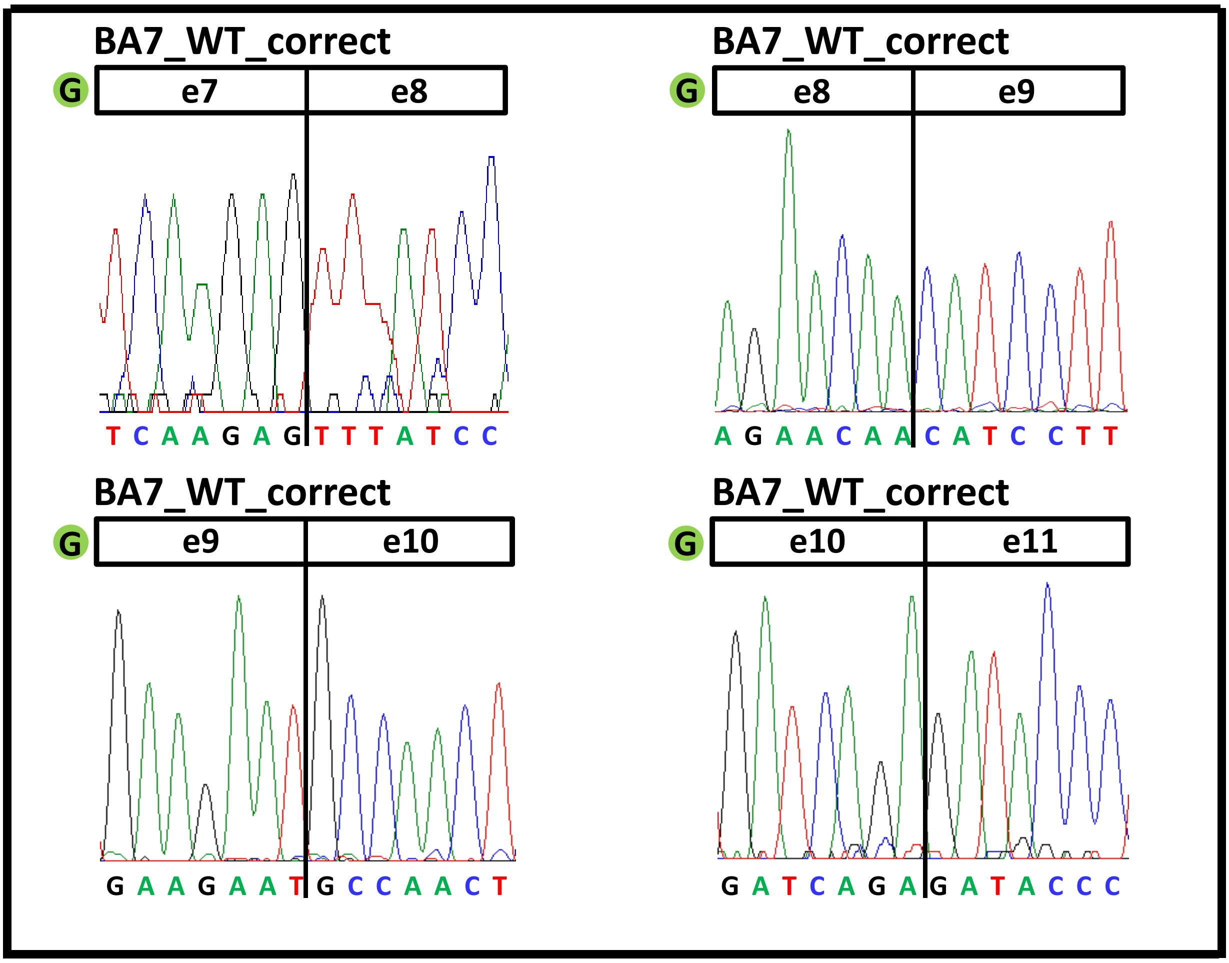
BA7_WT_correct
G
e7
e8
T C A A G A G T T T A T C C
BA7_WT_correct
G
e8
e9
A G A A C A A C A T C C T T
BA7_WT_correct
G
e9
e10
G A A G A A T G C C A A C T
BA7_WT_correct
G
e10
e11
G A T C A G A G A T A C C C

BA7

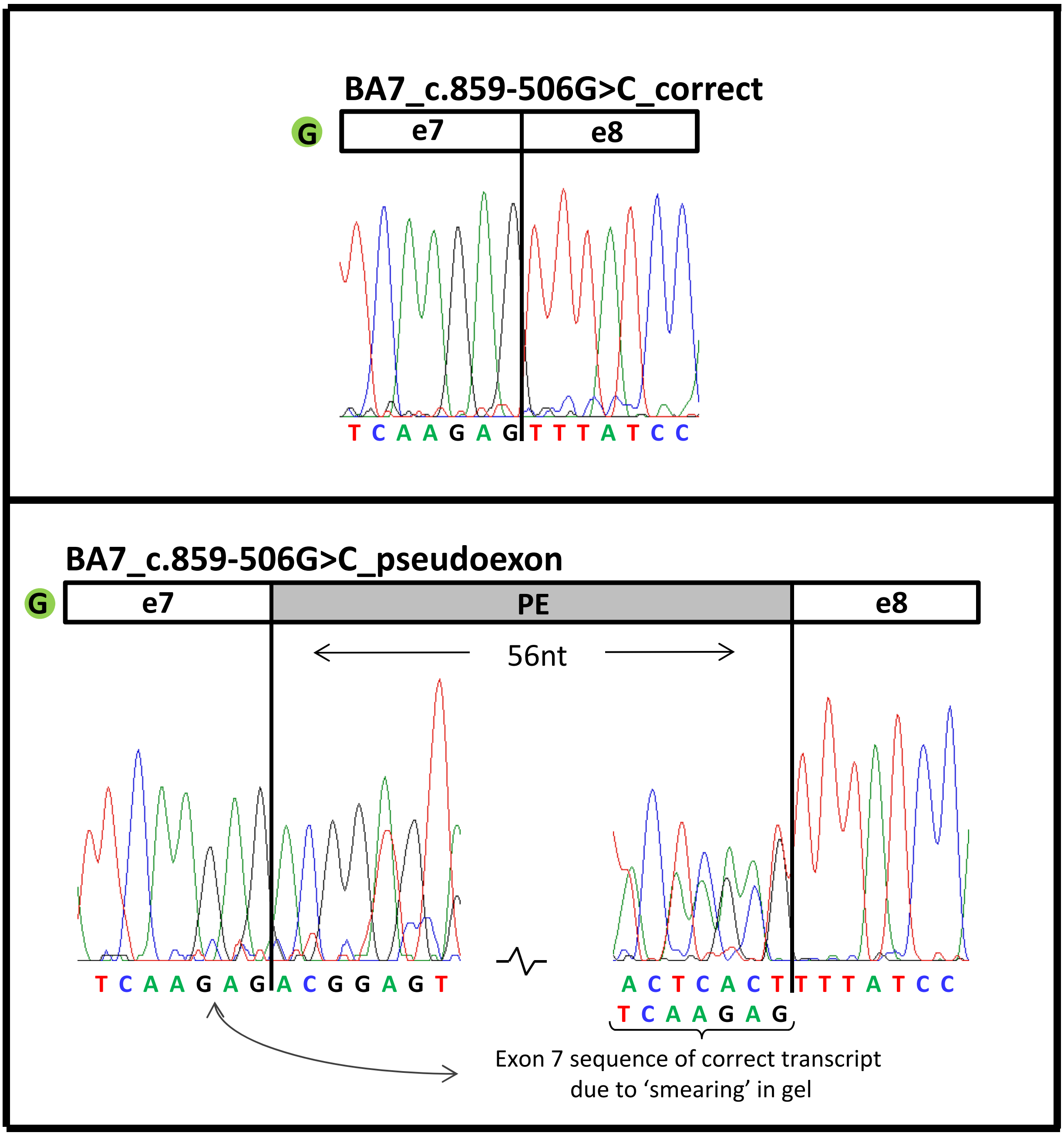

BA7_c.859-506G>C_correct
G
e7
e8
T C A A G A G T T T A T C C
BA7_c.859-506G>C_pseudoexon
G
e7
PE
e8
56nt
T C A A G A G A C G G A G T
A C T C A C T T T T A T C C
T C A A G A G
Exon 7 sequence of correct transcript
due to 'smearing' in gel

BA11

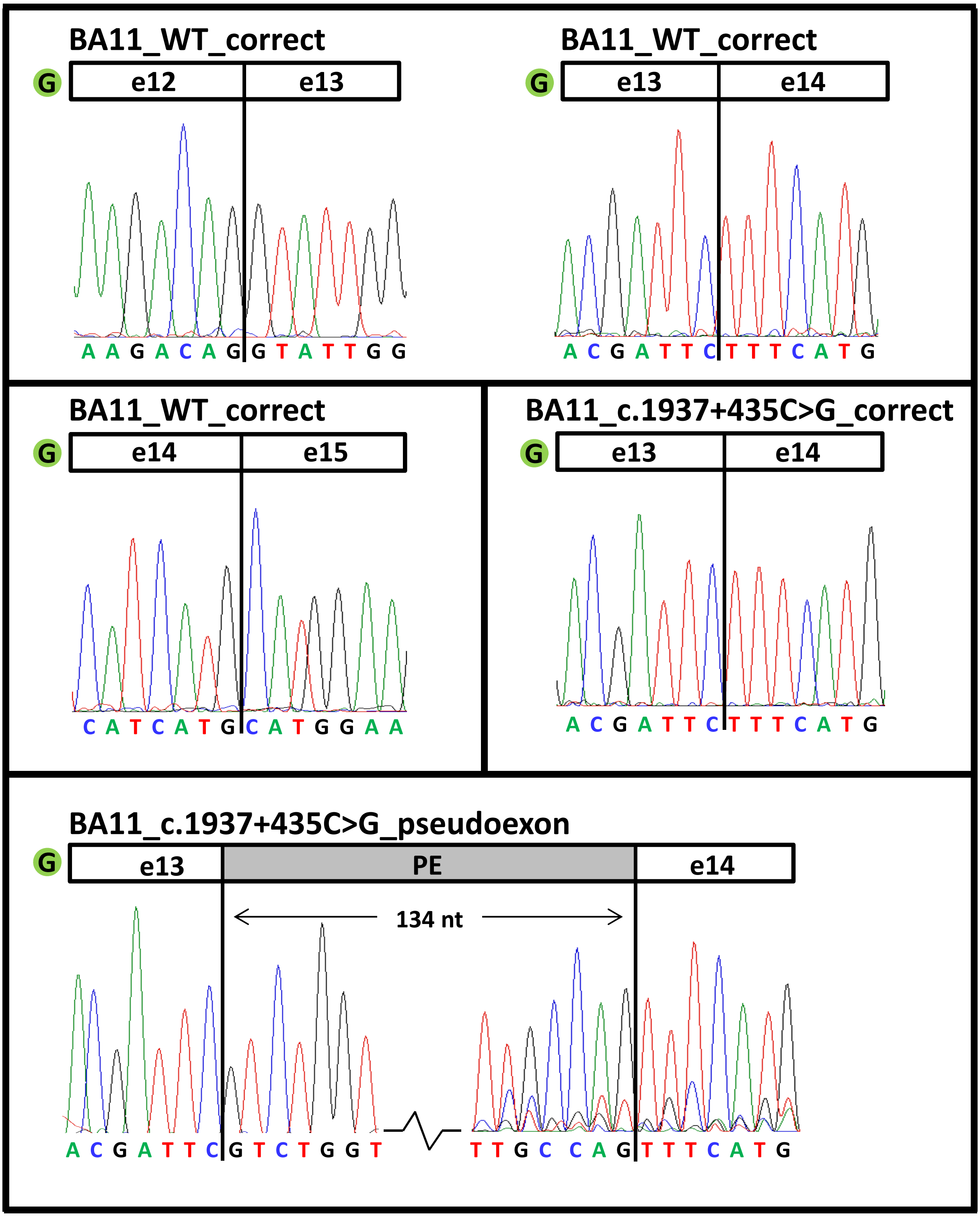

BA11_WT_correct
G
e12
e13
A A G A C A G G T A T T G G
BA11_WT_correct
G
e13
e14
A C G A T T C T T T C A T G
BA11_WT_correct
G
e14
e15
C A T C A T G C A T G G A A
BA11_c.1937+435C>G_correct
G
e13
e14
A C G A T T C T T T C A T G
BA11_c.1937+435C>G_pseudoexon
G
e13
PE
e14
134 nt
A C G A T T C G T C T G G T
T T G C C A G T T T C A T G

BA19

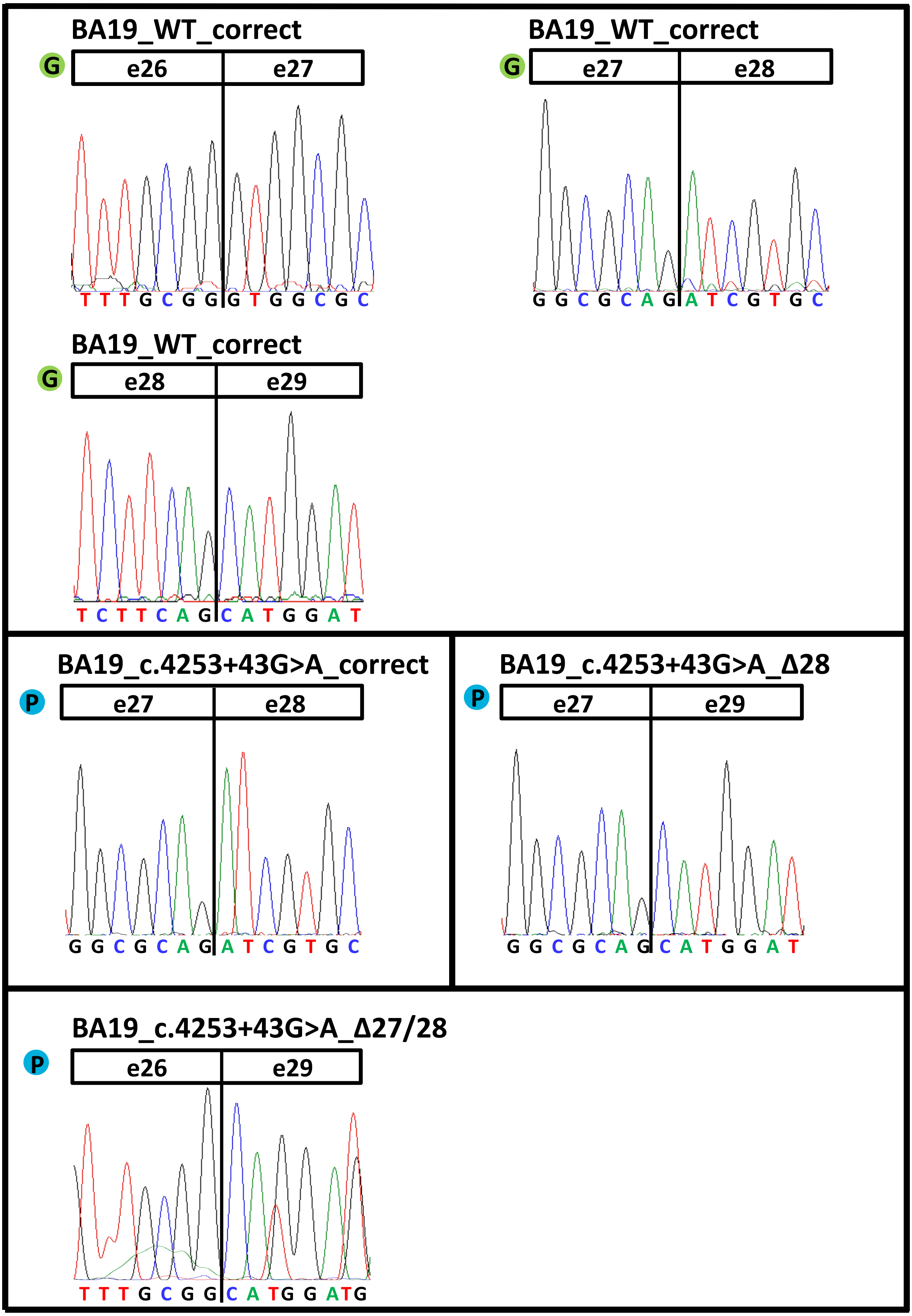

BA19_WT_correct
G
e26
e27
T T T G C G G G T G G C G C
BA19_WT_correct
G
e27
e28
G G C G C A G A T C G T G C
BA19_WT_correct
G
e28
e29
T C T T C A G C A T G G A T
BA19_c.4253+43G>A_correct
P
e27
e28
G G C G C A G A T C G T G C
BA19_c.4253+43G>A_Δ28
P
e27
e29
G G C G C A G C A T G G A T
BA19_c.4253+43G>A_Δ27/28
P
e26
e29
T T T G C G G C A TG G ATG

BA21

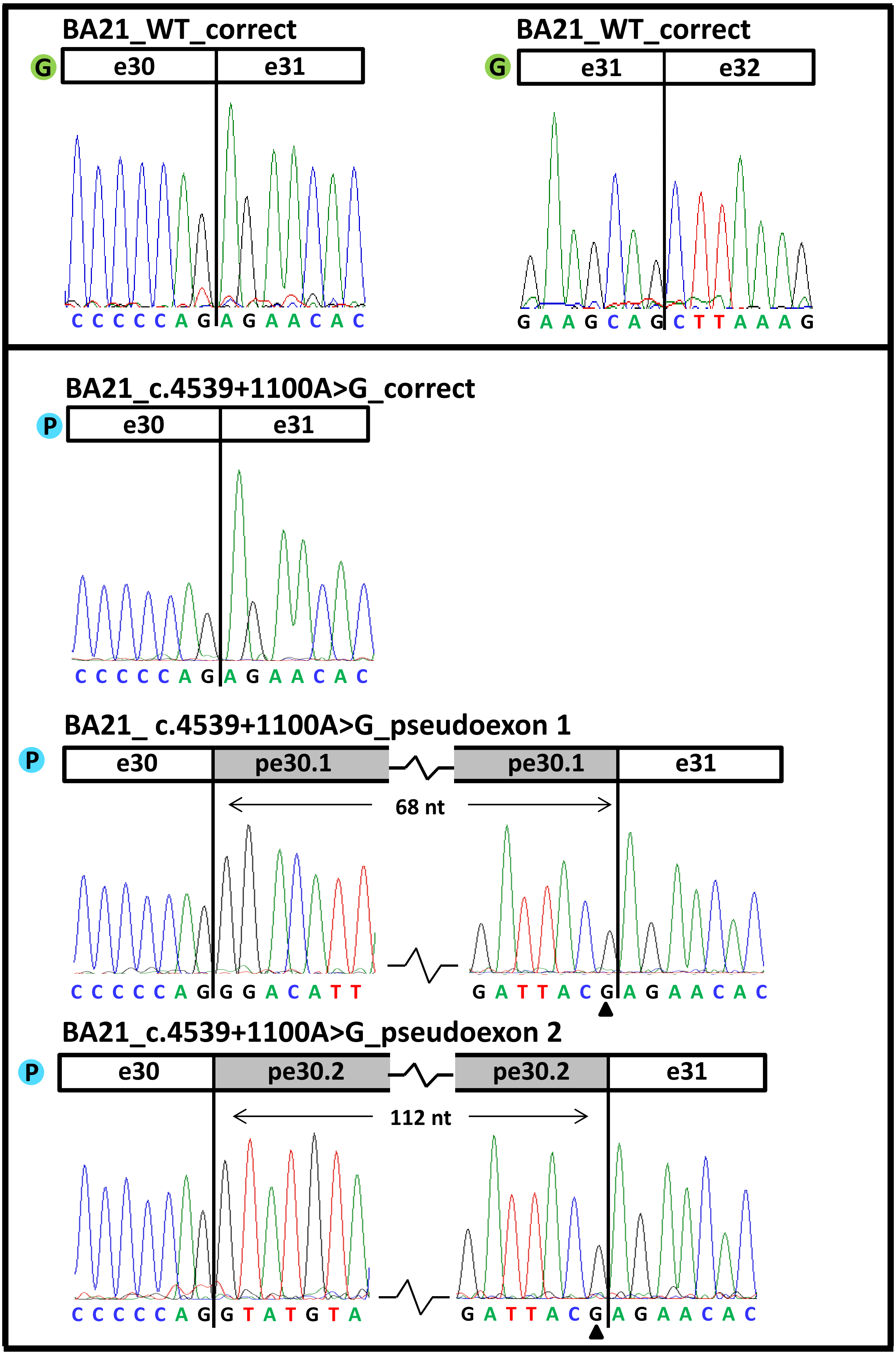
BA21_WT_correct
G
e30
e31
C C C C C A G A G A A C A C
BA21_WT_correct
G
e31
e32
G A A G C A G C T T A A A G
BA21_c.4539+1100A>G_correct
P
e30
e31
C C C C C A G A G A A C A C
BA21_ c.4539+1100A>G_pseudoexon 1
P
e30
pe30.1
pe30.1
e31
68 nt
C C C C C A G G G A C A T T
G A T T A C G A G A A C A C
BA21_c.4539+1100A>G_pseudoexon 2
P
e30
pe30.2
pe30.2
e31
112 nt
C C C C C A G G T A T G T A
G A T T A C G A G A A C A C

BA21

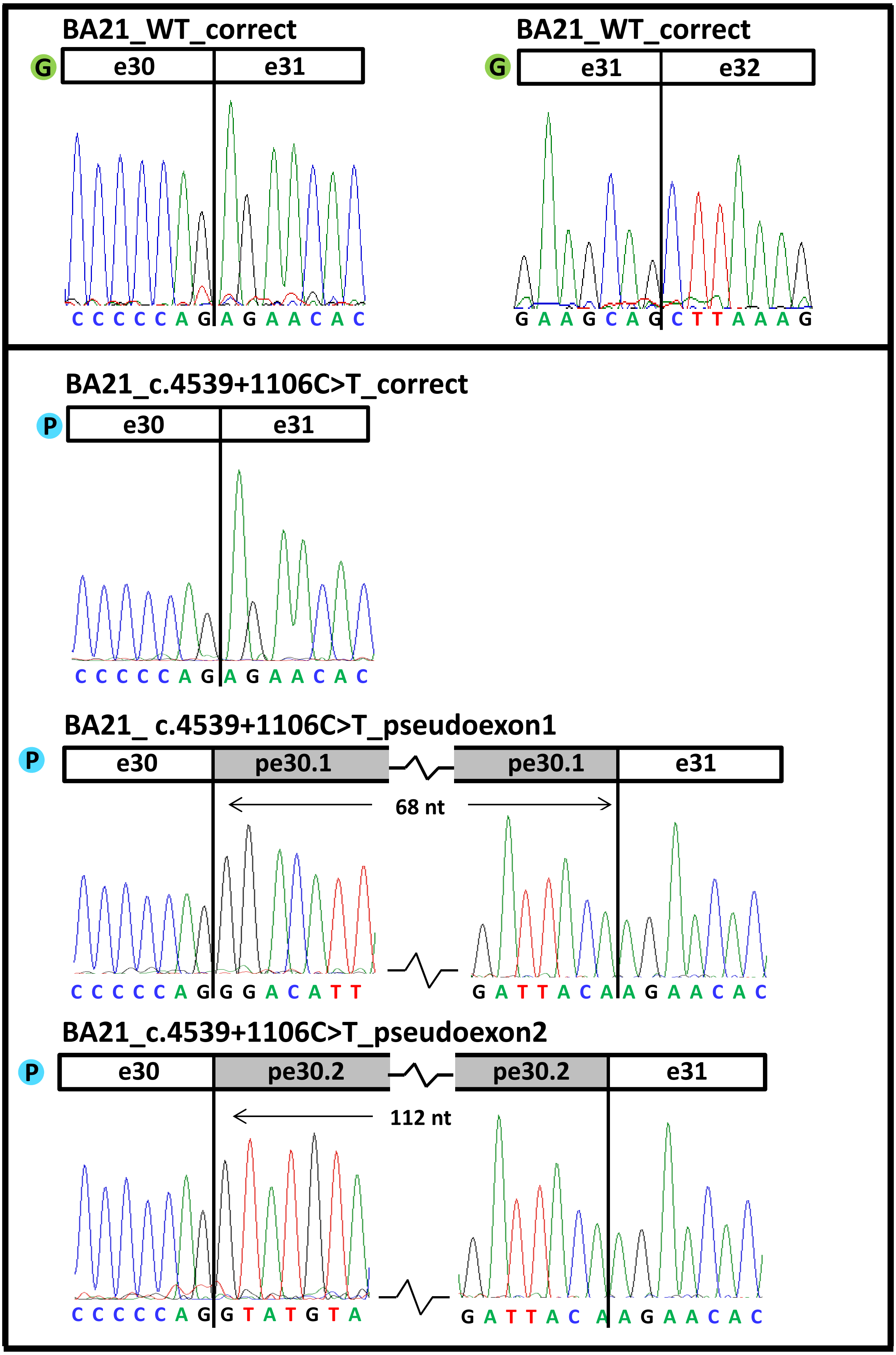
BA21_WT_correct
G
e30
e31
C C C C C A G A G A A C A C
BA21_WT_correct
G
e31
e32
G A A G C A G C T T A A A G
BA21_c.4539+1106C>T_correct
P
e30
e31
C C C C C A G A G A A C A C
BA21_ c.4539+1106C>T_pseudoexon1
P
e30
pe30.1
pe30.1
e31
68 nt
C C C C C A G G G A C A T T
G A T T A C A A G A A C A C
BA21_c.4539+1106C>T_pseudoexon2
P
e30
pe30.2
pe30.2
e31
112 nt
C C C C C A G G T A T G T A
G A T T A C A A G A A C A C

Fig. S3

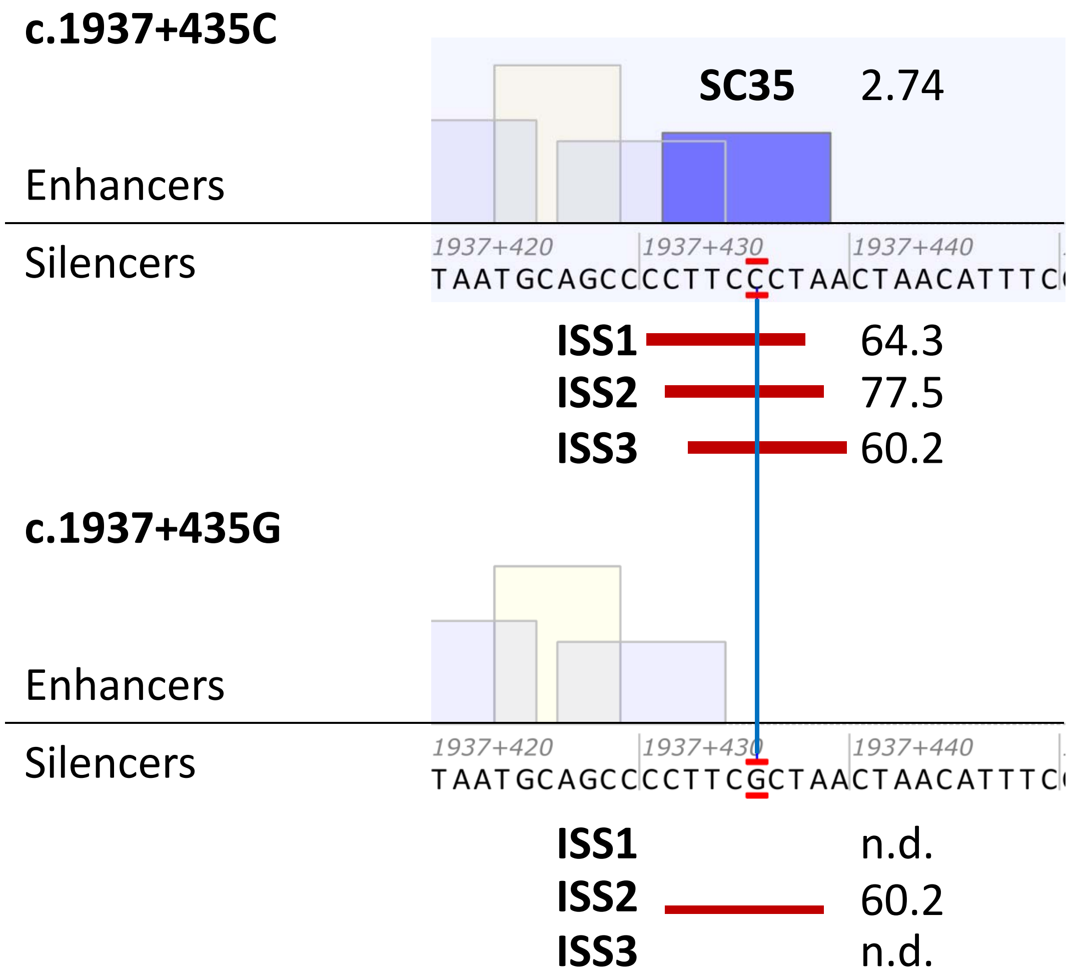

Fig. S3. Enhancer and silencer motif variation caused by c.1937+435C>G. The c.1937+435C>G variant disrupts one SC35 enhancer and two intronic splice silencers (ISS1 and ISS3), and reduces the strength of ISS2. n.d., not detected.

Fig. S4

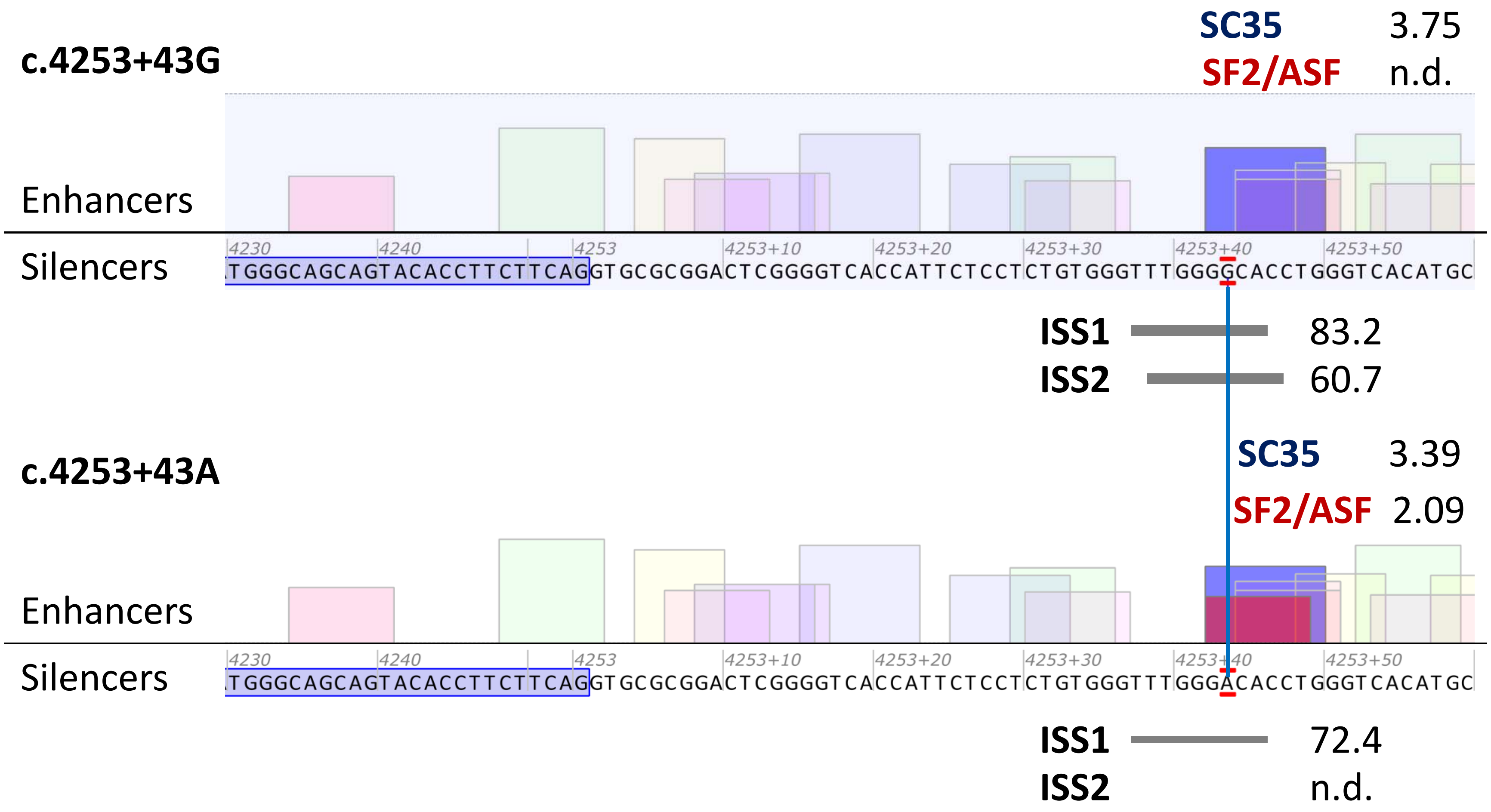

Fig. S4. Enhancer and silencer motif variation caused by c.4253+43G>A. The c.4253+43G>A variant weakens one SC35 and creates one SF2/ASF enhancer. It also weakens one intronic splice silencers (ISS1) and disrupts ISS2. n.d., not detected.

Pseudoexon exclusion

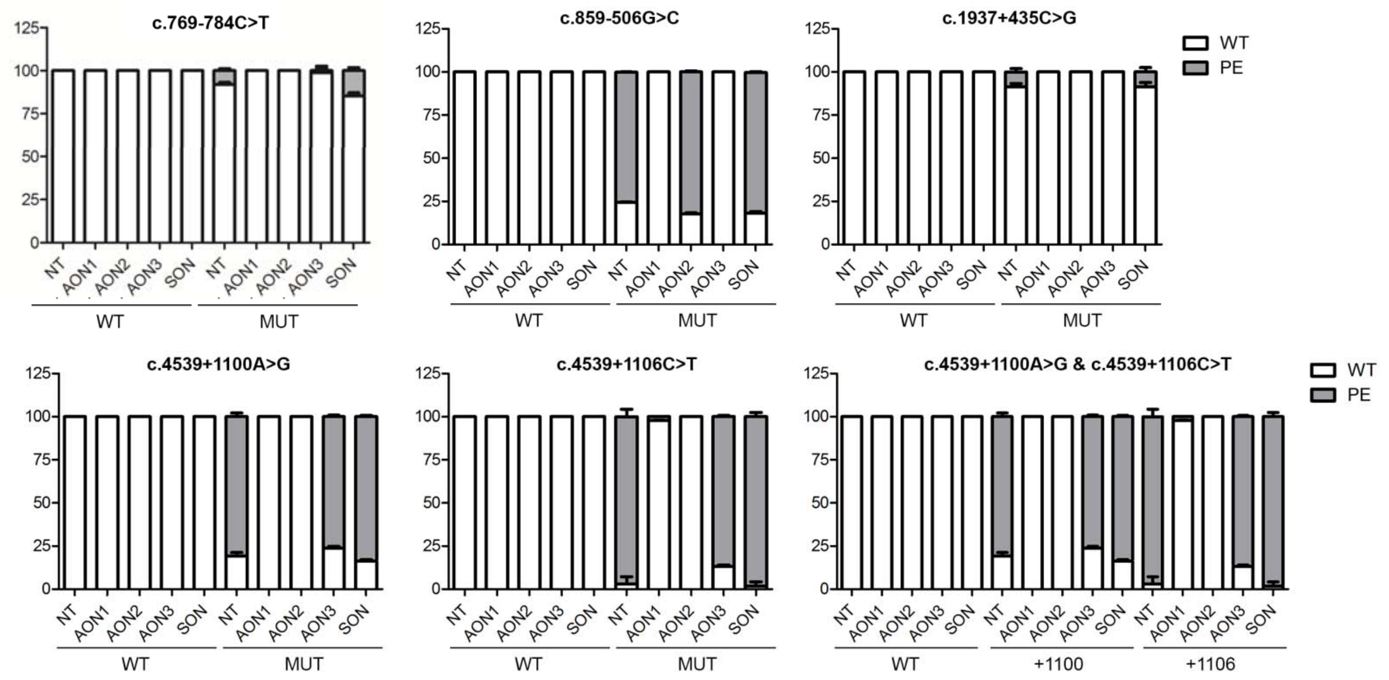

Exon inclusion

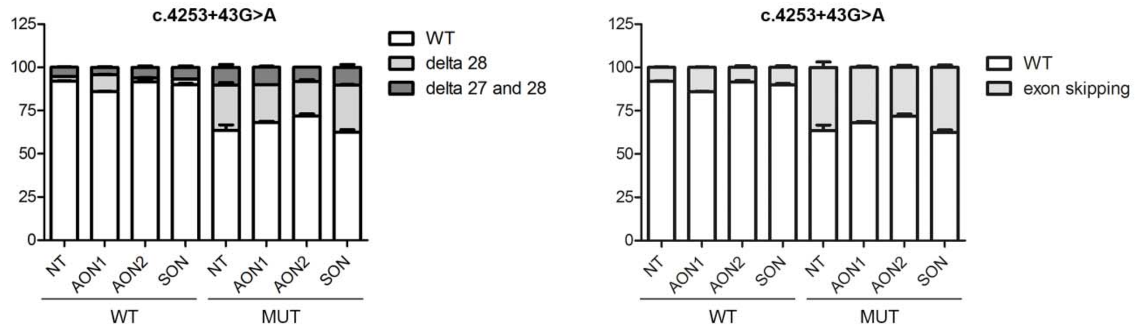

Fig. S5. Semiquantification by capillary analysis of the AON-based splicing correction in HEK293T. RT-PCR (n=2) were subjected to capillary analysis and the ratio between the wild-type (WT) and the pseudoexon (PE) peaks was calculated for each mutation using the WT and the mutant (MUT) construct. In the case of the c.4539+1100A>G and c.4539+1106C>T both PEs were counted as one to calculate the ratio. In addition, given that same AONs were used they were placed together in the same graph. For the exon inclusion approach the amount of exon skipping is depicted in two ways, the skipping of exon 28 and 27+28 separately or both counted as one exon skipping event. Error bars indicate standard deviation (s.d.).

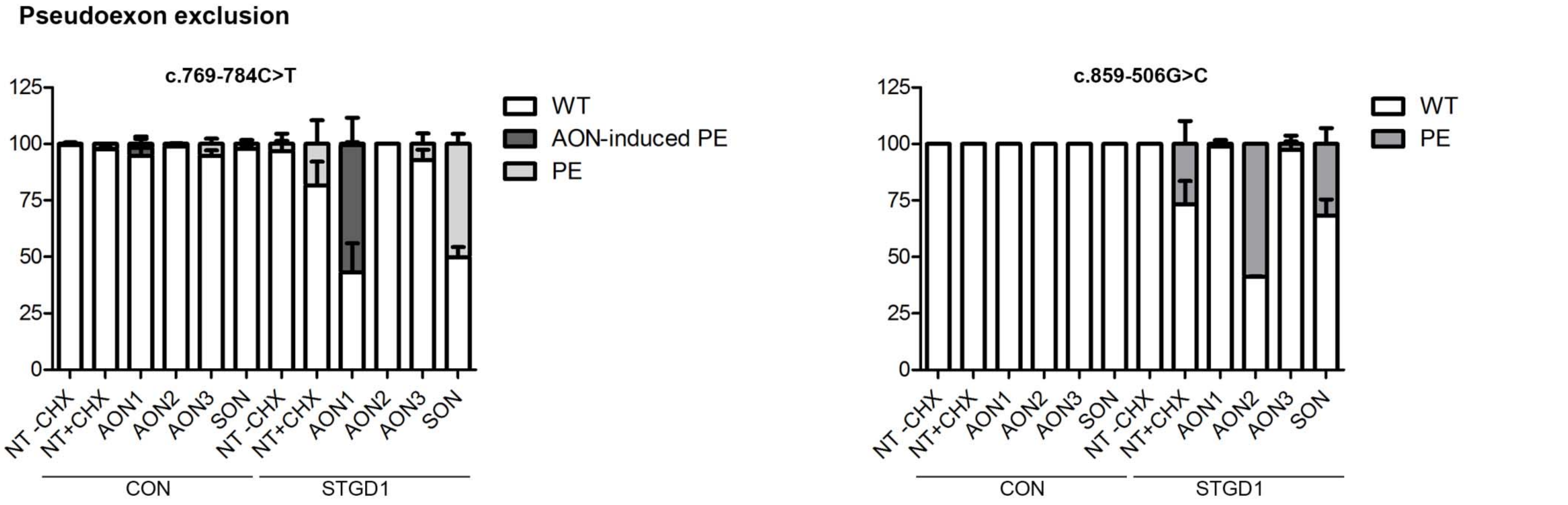

Fig. S6. Semiquantification by capillary analysis of the AON-based splicing correction in STGD1 individuals-derived fibroblast cells. The AON rescue was assessed by RT-PCR (n=2)and subsequently semi-quantified by capillary analysis. In this figure, the ratio between the pseudoexon (PE) and the wild-type (WT) peaks for two mutations is depicted both in control (CON) and STGD1-individual-derived (STGD1) fibroblast cells. For the c.769-784C>T variant a new PE was detected upon delivery of AON1 (AON-induced PE). This PE was the result of partial PE exclusion. Error bars represent the standard deviation (s.d.).

Table_S1. Primers for reverse transcription-PCR analysis. #, Rhodopsin exonic primer; *53, Rhodopsin 3' UTR; n.a., not applicable

Primer	Primer sequence (5'-3')	Genomic position (hg19)	cDNA position (hg19)	Product size WT band (bp)	Product size PE or skipping band (bp)	Experiment
RHO _exon 3_forward	CGGAGGTCAACAACGAGTCT	129251150#	587	134	296	Midigene
ABCA4 _exon 7_reverse	ACGGCTGTCTAGGAGTGTGG	94548974	792			
ABCA4 _exon7_forward	TCTGAGATCTTGGGGAGGAA	94548959	807	254	310	Midigene & Fibroblast
ABCA4 _exon8_reverse	TGGAGTCAATCCCCAGAAAG	94546073	1060			
ABCA4 _exon7_forward	TCTGAGATCTTGGGGAGGAA	94548959	807	748	n.a.	Midigene
ABCA4 _exon11_reverse	CTCCAGGTATTGATTGACCAG	94543246	1554			
*ABCA4*_ exon13_forward	GCCTATCTGCAGGACATGGT	94528245	1825	218	352	Midigene
*ABCA4*_ exon14_reverse	CGCAACTCCTTCTCCAAGAC	94526211	2042			
*ABCA4*_ exon26_forward	GAAGGTCACGGAGGATTCTG	94502336	3822	495	370	Midigene
*ABCA4*_ exon29_reverse	CCTGGCTTATTCAGGAGGAC	94496020	4316			
ABCA4 _exon30_forward	AAACATCACCCAGCTGTTCC	94495136	4404	257	325 & 369	Midigene
ABCA4 _exon32_reverse	TCATTGACCCAGAATTTGCTC	94488948	4661			
*ABCA4*_ exon42_forward	ATTTATCCAGGCACCTCCAG	94473853	5836	404	n.a.	Midigene
*ABCA4*_ exon45_reverse	GAGAGTTTCCGCTTGTTGC	94467457	6239			
RHO _exon5_forward	ATCTGCTGCGGCAAGAAC	129252475#	961	140	n.a.	Midigene
RHO _exon5_reverse	AGGTGTAGGGGATGGGAGAC	129252614#	*53			
ABCA4 _exon5_forward	GGAATACGAATAAGGGATATCTTG	94568693	448	354	516	Fibroblast
ABCA4 _exon7_reverse	ACGGCTGTCTAGGAGTGTGG	94548974	792			
ACTB _exon3_forward	ACTGGGACGACATGGAGAAG	5568922	233	382	n.a.	Fibroblast
ACTB _exon4_reverse	TCTCAGCTGTGGTGGTGAAG	5568099	615			

Table_S2. Antisense oligonucleotide sequences and characteristics

Variant	AON #	Sequence (5' → 3')	Length (nt)	T (°C)	%GC	Position relative to PE	Motif(s) targeted
c.769-784C>T	1	GAUGGAAUCACUGAUCCUAG	20	49.7	45	Acceptor site	SC35 and SRSF5
	2	AGCUCCAGAGACUGAUGUGA	20	51.8	50	PE	SC35, SRSF5 and SRSF6
	3	CUCACCACUGCUCCUGC	17	51.9	65	Donor site	SC35
c.859-506G>C	1	GACUGAGCAAUACUCCGUC	19	51.1	53	Acceptor site	SRSF5
	2	AUCACAGAGUGACCCCUAG	19	51.1	53	Acceptor site	Branch point, SRSF2 and SRSF5
	3	CUGAGCAAUACUCCGUCUG	19	51.1	53	Intronic	SRSF5 and covers the mutation
c.1937+435C>G	1	CUCCCAGGAACCAGACCUA	19	53.2	53	Acceptor site	SC35 ad SRSF5
	2	GCUCAUCCAACACAUUCCUC	20	51.8	58	PE	SRSF1 and SRSF5
	3	CCUGGGAUGGGAGUGUC	17	51.9	65	Donor site	SC35 and SRSF5
c.4253+43G>A	1	GACCCAGGUGUCCCAAACCCA	21	58.3	62	Intronic	NA and covers the mutation*
	2	AGCAUGUGACCCAGGUGUCCC	21	58.3	62	Intronic	NA and covers the mutation*
c.4539+1100A>G & c.4539+1106C>T	1	GUAAUCUGUUCUGGACUU	18	43.5	39	PE	SC35, SRSF5 and SRSF6
	2	UAGAACUCCCAGGACAGG	18	50.3	56	PE	SC35 and SRSF5
	3	CUAAAUCCCCCAGGAGAU	18	50.0	50	Intronic	SC35
All	SON	GAGGAAUGUGUUGGAUGAGC	20	51.8	50	PE	NA

T, melting temperature calculated using OligoCalc (http://biotools.nubic.northwestern.edu/OligoCalc.html).

*, these two AONs were designed to promote exon inclusion rather than skipping, thus ESEs were not assessed.

NA, not applicable; PE, pseudoexon

Table_S3. Variants found after targeted or whole genome sequencing of eight STGD1 cases carrying one causal variant and p.Asn1868Ile in *trans* , 25 mono-allelic STGD1 cases and three STGD1 cases with no *ABCA4* variant. HP, Haloplex-based targeted sequencing; WGS, whole genome sequencing

Sequencing method	Variant	Allele number	Sample ID
HP	c.66+37G>T	1	P2R7
HP	c.66+877G>A	1	P4R2_bis
HP	c.66+2316G>A	2	P9R1_bis, P5R6
HP	c.66+3257_66+3258insA	5	P7R5, P12R7, P1R5, P2R7, P5R7.
HP	c.67-3903_67-3902insAAGA	1	P2R1_bis
HP	c.67-3779T>G	2	P2R7, P10R1_bis
HP	c.67-3106T>C	2	P9R1_bis, P5R6
HP	c.67-2880C>A	1	P12R7
HP	c.67-619_67-613delCACAGTC	1	P5R6
WGS	c.442+793C>T	1	GC21017
HP	c.442+2578C>A	1	P8R4
HP	c.570+1310_570+1311insT	11	P12R7, P1R5, P2R2_bis, P2R7, P4R2_bis, P4R5, P5R3_bis, P5R7,P7R4, P7R6, P8R4
HP	c.768+771G>A	2	P3R1_bis, P12M6
WGS	c.768+1954G>A	1	GC17027
HP	c.768+2713T>A	1	P5R6
HP	c.768+4123G>C	1	P10R6
HP	c.768+7191_768+7202delTGTGTGTGTGTG	1	P4R6
HP	c.768+7329A>G	1	P12R7
HP	c.769-6917_769-6916insT	8	P9R6, P10R6, P2R2_bis, P3R1_bis, P7R4, P7R6, P8R1_bis, P8R4
HP	c.769-6030delA	1	P12R2_bis
HP	c.769-6030_769-6029insA	1	P10R2_bis
HP	c.769-5431G>A	1	P7R5
HP	c.769-5221T>A	1	P12R7
HP	c.769-2179G>A	1	P5R1_bis
HP	c.769-1331G>A	1	P7R2_bis
HP	c.769-784C>T	7	P7R4, P11R2_bis, P12R5, P2R7, P3R2_bis, P4R2_bis, P4R5
HP	c.858+526T>G	1	P12M6
HP	c.858+786A>G	1	P5R6
HP	c.859-550C>T	1	P12R7

HP	c.859-506G>C	3	P8R6, P1R3_bis, P5R7
HP	c.859-22T>C	1	P9R6
HP	c.1239+219A>G	1	P5R7
HP	c.1554+2495C>A	1	P12R7
HP	c.1554+3404A>T	1	P5R7
HP	c.1554+6237_1554+6238delCAinsTG	11	P12R5, P1R3_bis, P2R7, P3R2_bis, P4R2_bis, P4R5, P4R6, P5R3_bis, P5R7, P7R4, P8R6
HP	c.1554+6603A>G	1	P12R7
WGS	c.1555-6842A>G	1	GC17027
HP	c.1555-5641G>A	2	P12R2_bis, P12R5
HP	c.1555-5008C>T	1	P10R6
HP	c.1555-4901G>T	1	P8R1_bis
HP	c.1555-3782C>T	1	P10R6
HP	c.1555-2745A>G	2	P4R3_bis, P8R6
HP	c.1555-473_1555-472delAGinsT	1	P12R2_bis
HP	c.1937+435C>G	2	P7R6, P1R5
WGS	c.2160+207C>T	1	GC17027
HP	c.2587+1486G>A	1	P7R4
HP	c.2654-420C>T	1	P2R7
HP	c.2918+71delG	1	P12R7
HP	c.2918+472delAinsGTCTT	3	P3R2_bis, P7R5, P8R4
HP	c.2918+717C>T	1	P4R6
HP	c.2918+968C>G	2	P12R7, P1R5
HP	c.2919-576_2919-575delAA	3	P7R5, P7R2_bis, P10R2_bis
HP	c.3608-227C>T	1	P12R7
HP	c.3862+860C>T	1	P5R3_bis
HP	c.3862+1840T>C	1	P2R7
HP	c.4140G>A	1	P5R1_bis
HP/WGS	c.4253+43G>A	9	P9R6, P10R1_bis, P10R2_bis, P10R6, P12R2_bis, P2R2_bis, P7R2_bis, P7R5, GC20009
HP	c.4539+1100A>G	2	P12M6, P3R1_bis
WGS	c.4539+1106C>T	1	GC21017
HP	c.4539+1321A>G	3	P9R1_bis, P5R6, P6R6
HP	c.4539+2001G>A	1	P5R3_bis
HP	c.4540-1814T>G	1	P12R7
HP	c.4540-354C>T	1	P7R5
HP	c.4668-231C>T	1	P6R6

HP	c.5018+247T>C	1	P2R1_bis
HP	c.5018+547T>G	1	P2R7
HP	c.5196+1137G>A	1	P4R3_bis
HP	c.5197-491T>G	1	P8R1_bis
HP	c.5460+205_5460+206insA	11	P9R6, P10R6, P12M6, P12R5, P12R7, P2R2_bis, P2R7, P5R7, P7R4, P7R5, P8R4.
HP	c.5460+1315_5460+1316delGCinsTA	25	P9R6, P10R1_bis, P10R2_bis, P10R6, P12M6, P12R2_bis, P12R5, P12R7, P1R3_bis, P1R5, P2R1_bis, P2R2_bis, P2R7, P3R1_bis, P3R2_bis, P4R2_bis, P4R3_bis, P5R3_bis, P5R7, P6R1_bis, P7R2_bis, P7R4, P7R5, P7R6, P8R6
HP	c.5461-1353G>A	3	P8R6, P1R3_bis, P5R7
HP	c.5461-1295G>A	1	P10R6
HP	c.5461-355_5461-354delCGinsTA	23	P9R1_bis, P10R1_bis, P10R6, P11R2_bis, P12M6, P12R5, P1R5, P2R1_bis, P2R2_bis, P2R7, P3R1_bis, P3R2_bis, P4R2_bis, P4R5, P4R6, P5R1_bis, P5R6, P6R6, P7R2_bis, P7R4, P7R5, P8R1_bis, P8R4
HP	c.5838T>C	1	P5R6
HP	c.5843_5844delCAinsTG	1	P7R5
HP/WGS	c.6006-609T>A	8	P10R1_bis, P10R2_bis, P12R2_bis, P2R2_bis, P7R2_bis, P7R5, P9R6, GC20009
HP	c.6006-81G>A	1	P7R1_bis
HP/WGS	c.6147+677G>A	2	P12R7, GC17027
HP	c.6282+215T>G	5	P7R6, P10R2_bis, P1R3_bis, P3R2_bis, P4R2_bis
HP	c.6480-796delA	3	P12M6, P1R5, P5R6
HP	c.6480-604_6480-603insA	1	P8R6
HP	c.6480-346C>G	2	P10R6, P5R3_bis
HP	c.6816+440T>C	1	P8R4
HP	c.6817-1360T>C	1	P12R2_bis
HP	c.6817-850G>A	1	P9R6

Table_S3. Individual variants found after targeted or whole genome sequencing of eight STGD1 cases carrying one causal variant and p.Asn1868Ile in trans, 25 mono-allelic STGD1 cases and three STGD1 cases with no *ABCA4* variant. HP, Haloplex-based targeted sequencing; WGS, whole genome sequencing

Sequencing method	Variant	Allele number	Sample ID
HP	c.1239+219A>G	1	P5R7
HP	c.1554+2495C>A	1	P12R7
HP	c.1554+3404A>T	1	P5R7
HP	c.1554+6237_1554+6238delCAinsTG	11	P12R5, P1R3_bis, P2R7, P3R2_bis, P4R2_bis, P4R5, P4R6, P5R3_bis, P5R7, P7R4, P8R6
HP	c.1554+6603A>G	1	P12R7
HP	c.1555-2745A>G	2	P4R3_bis, P8R6
HP	c.1555-3782C>T	1	P10R6
HP	c.1555-473_1555-472delAGinsT	1	P12R2_bis
HP	c.1555-4901G>T	1	P8R1_bis
HP	c.1555-5008C>T	1	P10R6
HP	c.1555-5641G>A	2	P12R2_bis, P12R5
WGS	c.1555-6842A>G	1	GC17027
HP	c.1937+435C>G	2	P7R6, P1R5
WGS	c.2160+207C>T	1	GC17027
HP	c.2587+1486G>A	1	P7R4
HP	c.2654-420C>T	1	P2R7
HP	c.2918+472delAinsGTCTT	3	P3R2_bis, P7R5, P8R4
HP	c.2918+717C>T	1	P4R6
HP	c.2918+71delG	1	P12R7
HP	c.2918+968C>G	2	P12R7, P1R5
HP	c.2919-576_2919-575delAA	3	P7R5, P7R2_bis, P10R2_bis
HP	c.3608-227C>T	1	P12R7
HP	c.3862+1840T>C	1	P2R7
HP	c.3862+860C>T	1	P5R3_bis
HP	c.4140G>A	1	P5R1_bis
HP/WGS	c.4253+43G>A	9	P9R6, P10R1_bis, P10R2_bis, P10R6, P12R2_bis, P2R2_bis, P7R2_bis, P7R5, GC20009
WGS	c.442+793C>T	1	GC21017
HP	c.442+2578C>A	1	P8R4

HP	c.4539+1100A>G	2	P12M6, P3R1_bis
WGS	c.4539+1106C>T	1	GC21017
HP	c.4539+1321A>G	3	P9R1_bis, P5R6, P6R6
HP	c.4539+2001G>A	1	P5R3_bis
HP	c.4540-1814T>G	1	P12R7
HP	c.4540-354C>T	1	P7R5
HP	c.4668-231C>T	1	P6R6
HP	c.5018+247T>C	1	P2R1_bis
HP	c.5018+547T>G	1	P2R7
HP	c.5196+1137G>A	1	P4R3_bis
HP	c.5197-491T>G	1	P8R1_bis
HP	c.5460+1315_5460+1316delGCinsTA	25	P9R6, P10R1_bis, P10R2_bis, P10R6, P12M6, P12R2_bis, P12R5, P12R7, P1R3_bis, P1R5, P2R1_bis, P2R2_bis, P2R7, P3R1_bis, P3R2_bis, P4R2_bis, P4R3_bis, P5R3_bis, P5R7, P6R1_bis, P7R2_bis, P7R4, P7R5, P7R6, P8R6,
HP	c.5460+205_5460+206insA	11	P9R6, P10R6, P12M6, P12R5, P12R7, P2R2_bis, P2R7, P5R7, P7R4, P7R5, P8R4.
HP	c.5461-1295G>A	1	P10R6
HP	c.5461-1353G>A	3	P8R6, P1R3_bis, P5R7
HP	c.5461-355_5461-354delCGinsTA	23	P9R1_bis, P10R1_bis, P10R6, P11R2_bis, P12M6, P12R5, P1R5, P2R1_bis, P2R2_bis, P2R7, P3R1_bis, P3R2_bis, P4R2_bis, P4R5, P4R6, P5R1_bis, P5R6, P6R6, P7R2_bis, P7R4, P7R5, P8R1_bis, P8R4
HP	c.570+1310_570+1311insT	11	P12R7, P1R5, P2R2_bis, P2R7, P4R2_bis, P4R5, P5R3_bis, P5R7,P7R4, P7R6, P8R4
HP	c.5838T>C	1	P5R6
HP	c.5843_5844delCAinsTG	1	P7R5
HP/WGS	c.6006-609T>A	8	P10R1_bis, P10R2_bis, P12R2_bis, P2R2_bis, P7R2_bis, P7R5, P9R6, GC20009
HP	c.6006-81G>A	1	P7R1_bis
HP/WGS	c.6147+677G>A	2	P12R7, GC17027
HP	c.6282+215T>G	5	P7R6, P10R2_bis, P1R3_bis, P3R2_bis, P4R2_bis
HP	c.6480-346C>G	2	P10R6, P5R3_bis
HP	c.6480-604_6480-603insA	1	P8R6
HP	c.6480-796delA	3	P12M6, P1R5, P5R6
HP	c.66+2316G>A	2	P9R1_bis, P5R6
HP	c.66+3257_66+3258insA	5	P7R5, P12R7, P1R5, P2R7, P5R7.
HP	c.66+37G>T	1	P2R7
HP	c.66+877G>A	1	P4R2_bis
HP	c.67-2880C>A	1	P12R7

HP	c.67-3106T>C	2	P9R1_bis, P5R6
HP	c.67-3779T>G	2	P2R7, P10R1_bis
HP	c.67-3903_67-3902insAAGA	1	P2R1_bis
HP	c.67-619_67-613delCACAGTC	1	P5R6
HP	c.6816+440T>C	1	P8R4
HP	c.6817-1360T>C	1	P12R2_bis
HP	c.6817-850G>A	1	P9R6
HP	c.768+2713T>A	1	P5R6
HP	c.768+4123G>C	1	P10R6
HP	c.768+7191_768+7202delTGTGTGTGTGTG	1	P4R6
HP	c.768+7329A>G	1	P12R7
HP	c.768+771G>A	2	P3R1_bis, P12M6
WGS	c.768+1954G>A	1	GC17027
HP	c.769-1331G>A	1	P7R2_bis
HP	c.769-2179G>A	1	P5R1_bis
HP	c.769-5221T>A	1	P12R7
HP	c.769-5431G>A	1	P7R5
HP	c.769-6030_769-6029insA	1	P10R2_bis
HP	c.769-6030delA	1	P12R2_bis
HP	c.769-6917_769-6916insT	8	P9R6, P10R6, P2R2_bis, P3R1_bis, P7R4, P7R6, P8R1_bis, P8R4
HP	c.769-784C>T	7	P7R4, P11R2_bis, P12R5, P2R7, P3R2_bis, P4R2_bis, P4R5
HP	c.858+526T>G	1	P12M6
HP	c.858+786A>G	1	P5R6
HP	c.859-22T>C	1	P9R6
HP	c.859-506G>C	3	P8R6, P1R3_bis, P5R7
HP	c.859-550C>T	1	P12R7

Table_S4. Splice prediction analysis of 11 candidate non-coding variants. Non-coding variants were selected for splice assays if they were located at putative splice sites with a relative strength of at least 75% of the maximal score [0-max] in at least two out of five splice prediction programs, and which led to an increased splice prediction scores of at least 2% compared to the wild-type (wt) (green cells). *Variants selected because of their recurrency in the 45 genetically unsolved STGD1 cases. #Variant selected because located in the genomic sequence of an alternate exon 13 (Chr1: 94,527,737-94,527,644) that was previously described (Braun et al. Hum Mol Genet 22:5136-5145, 2013).
n.a., not applicable

**Variant**	localSS_wt_SSF score [0-100]	localSS_var_SSF score [0-100]	ΔlocalSS_SSF	localSS_wt_MaxEntScan score [0-12]	localSS_var_MaxEnt Scan score [0-12]	ΔlocalSS_MaxEnt Scan	localSS_wt_NNSplice score [0-1]	localSS_var_NNSplice score [0-1]	ΔlocalSS_NN Splice	localSS_wt_GeneSplicer score [0-15]	localSS_var_GeneSplicer score [0-15]	ΔlocalSS_GeneSplicer	localSS_wt_HSF score [0-100]	localSS_var_HSF score [0-100]	ΔlocalSS_HSF
**c.768+7329A>G**	70.9	83.0	12.1	n.a.	7.8	7.8	0.1	1.0	0.9	n.a.	2.4	2.4	75.8	88.0	12.2
**c.769-784C>T***	76.8	78.9	2.1	6.6	6.9	0.3	0.5	0.5	0.0	2.9	3.5	0.6	82.9	82.2	-0.7
**c.858+526T>G**	n.a.	75.5	75.5	n.a.	4.1	4.1	n.a	0.7	0.7	n.a	1.9	1.9	n.a	83.4	83.4
**c.859-506G>C**	78.7	89.5	10.8	0.8	8.1	7.3	0.0	1.0	1.0	1.5	12.0	10.5	77.8	88.1	10.3
**c.1555-5008C>T**	76.9	81.0	4.1	7.7	8.7	1.0	0.8	0.9	0.1	2.3	4.2	1.9	79.2	81.0	1.8
**c.1937+435C>G#**	n.a.	38.2	38.2	n.a.	n.a.	n.a.	n.a.	n.a.	n.a.	n.a.	n.a.	n.a.	n.a.	n.a.	n.a.
**c.4253+43G>A***	62.7	n.a.	-62.7	n.a.	n.a.	n.a.	n.a.	n.a.	n.a.	n.a.	n.a.	n.a.	n.a.	n.a.	n.a.
**c.4539+1100A>G**	68.5	80.6	12.1	4.7	9.4	4.7	0.4	1.0	0.6	3.3	7.8	4.5	80.9	91.0	10.1
**c.4539+1106C>T**	68.5	74.3	5.8	4.7	8.3	3.6	0.4	0.9	0.5	3.3	6.4	3.1	80.4	82.6	2.2
**c.6006-609T>A***	82.4	82.4	0.0	7.9	7.9	0.0	1.0	1.0	0.0	5.8	6.2	0.4	82.0	82.0	0.0
**c.6148-421T>C**	85.7	89.8	4.1	7.8	9.8	2.0	1.0	1.0	0.0	7.7	10.5	2.8	89.2	91.2	2.0

Table_S5. Gateway-tagged *ABCA4* primers to amplify BA30 genomic segment, primers for sequence validation and sequence variants found.
*= entry clone forward primer; **= entry clone reverse primer; n.a., not applicable.

Wild-type fragment	Primer sequence with TAGS (5'-3')	Primer sequence without TAGS (5'-3')	Genomic position (hg 19)	cDNA position (hg 19)	Insert size (bp)
BA30_forward	GGGGACAAGTTTGTACAAAAAAGCAGGCTTCacagggcctcagtgagaatc	ACAGGGCCTCAGTGAGAATC	94474098	5835+209	7127
BA30_reverse	GGGGACCACTTTGTACAAGAAAGCTGGGTGaggtcactcaggtcacttcc	AGGTCACTCAGGTCACTTCC	94466972	6283-311	

BA_ID	Primer number	Sequence (5'-3')	Intron/ Exon	Forward/ Reverse	Genomic position (hg19)	cDNA position (hg19)
BA30	6147*	TCGCGTTAACGCTAGCATGGATCTC				
BA30	6148**	GTAACATCAGAGATTTTGAGACAC				
BA30	71168	ACCCTACTTGCTTCCCTGAG	intron 43	forward	94471880	6006-742
BA30	71170	ACTGGCTCCTCTTTCATTGC	intron 43	forward	94471581	6006-443
BA30	75753	TTACCTTTATGCCCGGCTTC	exon 44	forward	94471045	6099
BA30	75754	AGGTCATGGATAGGAACCGG	intron 44	forward	94469843	6147+1154
BA30	75755	CGGAAGACAGATACACACATGC	intron 44	reverse	94469697	6147+1300
BA30	75756	AGTGAGCTGTGATCAAGCC	intron 44	forward	94469266	6148**-1718

BA ID	Genomic position (hg19)	cDNA position (hg19)	Reference	Variant	db SNP ID	Variation	Minor Allele Frequency/ Minor Allele Count
BA30	94474091	5835+216	C	_	n.a.		
BA30	94474020	5836-167	C	T	rs1191234	G/A	G = 0.109/545
BA30	94472517	6005+673	G	A	n.a.	C/T	
BA30	94472489	6005+701	A	G	rs557026	T/C	T = 0.11/550
BA30	94471075	6069	T	C	rs1762114	A/G	A = 0.23/1152

Table S6: Persons with genetically unsolved STGD1

Patient_ID	Gender	Age at onset (yrs)	*ABCA4*_Allele1 DNA	*ABCA4*_Allele1 protein
**I**	M	21	c.5882G>A	p.(Gly1961Glu)
**II**	F	20	c.(2588G>C)	p.[Gly863Ala, Gly863del]
**III**	F	31	c.[5461-10T>C; 5603A>T]	p.[Thr1821Valfs*13, Thr1821Aspfs*6; Asn1868Ile]
**IV**	M	24	c.[5461-10T>C; 5603A>T]	p.[Thr1821Valfs*13, Thr1821Aspfs*6; Asn1868Ile]
**V**	M	10	c.[5461-10T>C; 5603A>T]	p.[Thr1821Valfs*13, Thr1821Aspfs*6; Asn1868Ile]
**VI**	M	10	c.5714+5G>A	p.[=, Glu1863Leufs*33]
**VII**	M	45	c.[5461-10T>C; 5603A>T]	p.[Thr1821Valfs*13, Thr1821Aspfs*6; Asn1868Ile]
**VIII**	M	63*	c.[5461-10T>C; 5603A>T]	p.[Thr1821Valfs*13, Thr1821Aspfs*6; Asn1868Ile]
**IX**	M	40*	c.5882G>A	p.(Gly1961Glu)
**X**	M	55*	c.[2588G>C; 5603A>T]	p.[Gly863Ala, Gly863del; Asn1868Ile]
**XI**	n.a.	32*	+	+
**XII**	n.a.	51*	c.6445C>T	p.(Arg2149*)

*Unknown age of onset, current age; n.a., not assessed; +, wild-type.

Table_S7. Capillary fragment analyzer results for RT-PCR products of six non-treated (NT) or treated with two or three different AONs or sense oligonucleotide (AON1, AON2, AON3, SON) wild-type and mutant midigenes. For five variants, AONs were designed to induce pseudoexon exclusion, while for c.4253+43G>A to induce exon inclusion. Two independent experiments were performed followed by two RT-PCR reactions per experiment, products were run onto a capillary electrophoresis system to quantify the different fragment quantities expressed as percent (%) of the entire fragment mix. SD, standard deviation.

Pseudoexon exclusion – fragment analyzer results

c.769-784C>T

Band	WT midigene										Mutant midigene									
	NT		AON1		AON2		AON3		SON		NT		AON1		AON2		AON3		SON	
	%	SD	%	SD	%	SD	%	SD	%	SD	%	SD	%	SD	%	SD	%	SD	%	SD
WT	100.00%	0.00%	100.00%	0.00%	100.00%	0.00%	100.00%	0.00%	100.00%	0.00%	91.32%	1.19%	100.00%	0.00%	100.00%	0.00%	98.06%	2.61%	85.90%	1.88%
PE	0.00%	0.00%	0.00%	0.00%	0.00%	0.00%	0.00%	0.00%	0.00%	0.00%	7.97%	1.19%	0.00%	0.00%	0.00%	0.00%	1.17%	2.61%	14.10%	1.88%

c.859-506G>C

Band	WT midigene										Mutant midigene									
	NT		AON1		AON2		AON3		SON		NT		AON1		AON2		AON3		SON	
	%	SD	%	SD	%	SD	%	SD	%	SD	%	SD	%	SD	%	SD	%	SD	%	SD
WT	100.00%	0.00%	100.00%	0.00%	100.00%	0.00%	100.00%	0.00%	100.00%	0.00%	24.44%	0.32%	100.00%	0.00%	18.25%	1.18%	100.00%	0.00%	18.13%	0.94%
PE	0.00%	0.00%	0.00%	0.00%	0.00%	0.00%	0.00%	0.00%	0.00%	0.00%	75.56%	0.32%	0.00%	0.00%	81.75%	1.18%	0.00%	0.00%	81.87%	0.94%

c.1937+435C>G

Band	WT midigene										Mutant midigene									
	NT		AON1		AON2		AON3		SON		NT		AON1		AON2		AON3		SON	
	%	SD	%	SD	%	SD	%	SD	%	SD	%	SD	%	SD	%	SD	%	SD	%	SD
WT	100.00%	0.00%	100.00%	0.00%	100.00%	0.00%	100.00%	0.00%	100.00%	0.00%	91.37%	1.95%	100.00%	0.00%	100.00%	0.00%	100.00%	0.00%	91.31%	2.56%
PE	0.00%	0.00%	0.00%	0.00%	0.00%	0.00%	0.00%	0.00%	0.00%	0.00%	8.63%	1.95%	0.00%	0.00%	0.00%	0.00%	0.00%	0.00%	8.69%	2.56%

c.4539+1100A>G

Band	WT midigene										Mutant midigene									
	NT		AON1		AON2		AON3		SON		NT		AON1		AON2		AON3		SON	
	%	SD	%	SD	%	SD	%	SD	%	SD	%	SD	%	SD	%	SD	%	SD	%	SD
WT	100.00%	0.00%	100.00%	0.00%	100.00%	0.00%	100.00%	0.00%	100.00%	0.00%	19.19%	2.12%	100.00%	0.00%	100.00%	0.00%	23.92%	0.88%	16.43%	0.78%
PE1	0.00%	0.00%	0.00%	0.00%	0.00%	0.00%	0.00%	0.00%	0.00%	0.00%	60.65%	0.80%	0.00%	0.00%	0.00%	0.00%	50.67%	1.23%	65.40%	3.04%
PE2	0.00%	0.00%	0.00%	0.00%	0.00%	0.00%	0.00%	0.00%	0.00%	0.00%	20.15%	1.32%	0.00%	0.00%	0.00%	0.00%	25.41%	2.11%	18.17%	2.26%

c.4539+1100A>G (both exon skipping as one event)

Band	WT midigene										Mutant midigene									
	NT		AON1		AON2		AON3		SON		NT		AON1		AON2		AON3		SON	
	%	SD	%	SD	%	SD	%	SD	%	SD	%	SD	%	SD	%	SD	%	SD	%	SD
WT	100.00%	0.00%	100.00%	0.00%	100.00%	0.00%	100.00%	0.00%	100.00%	0.00%	19.19%	2.12%	100.00%	0.00%	100.00%	0.00%	23.92%	0.88%	16.43%	0.78%
PE1+PE2	0.00%	0.00%	0.00%	0.00%	0.00%	0.00%	0.00%	0.00%	0.00%	0.00%	80.81%	2.12%	0.00%	0.00%	0.00%	0.00%	76.08%	0.88%	83.57%	0.78%

c.4539+1106C>T

Band	WT midigene										Mutant midigene									
	NT		AON1		AON2		AON3		SON		NT		AON1		AON2		AON3		SON	
	%	SD	%	SD	%	SD	%	SD	%	SD	%	SD	%	SD	%	SD	%	SD	%	SD
WT	100.00%	0.00%	100.00%	0.00%	100.00%	0.00%	100.00%	0.00%	100.00%	0.00%	3.02%	4.27%	97.88%	0.13%	100.00%	0.00%	13.30%	0.66%	1.75%	2.48%
PE1	0.00%	0.00%	0.00%	0.00%	0.00%	0.00%	0.00%	0.00%	0.00%	0.00%	93.12%	4.29%	0.00%	0.00%	0.00%	0.00%	75.99%	2.01%	97.09%	4.11%
PE2	0.00%	0.00%	0.00%	0.00%	0.00%	0.00%	0.00%	0.00%	0.00%	0.00%	3.87%	0.02%	2.12%	0.13%	0.00%	0.00%	10.71%	1.35%	1.16%	1.63%

c.4539+1106C>T (both exon skipping as one event)

Band	WT midigene										Mutant midigene									
	NT		AON1		AON2		AON3		SON		NT		AON1		AON2		AON3		SON	
	%	SD	%	SD	%	SD	%	SD	%	SD	%	SD	%	SD	%	SD	%	SD	%	SD
WT	100.00%	0.00%	100.00%	0.00%	100.00%	0.00%	100.00%	0.00%	100.00%	0.00%	3.02%	4.27%	97.88%	0.13%	100.00%	0.00%	13.30%	0.66%	1.75%	2.48%
PE1+PE2	0.00%	0.00%	0.00%	0.00%	0.00%	0.00%	0.00%	0.00%	0.00%	0.00%	96.98%	4.27%	2.12%	0.13%	0.00%	0.00%	86.70%	0.66%	98.25%	2.48%

Exon inclusion – fragment analyzer results

c.4253+43G>A

Band	WT midigene								Mutant midigene							
	NT		AON1		AON2		SON		NT		AON1		AON2		SON	
	%	SD	%	SD	%	SD	%	SD	%	SD	%	SD	%	SD	%	SD
delta27+28	5.32%	0.39%	4.29%	0.39%	6.14%	0.84%	6.86%	0.75%	10.38%	1.63%	10.16%	0.71%	8.32%	0.03%	10.25%	1.62%
delta28	2.80%	0.01%	9.80%	0.13%	2.39%	0.11%	3.34%	0.13%	26.15%	1.59%	21.82%	0.06%	19.81%	1.20%	27.19%	0.31%
WT	91.88%	0.38%	85.91%	0.26%	91.47%	0.95%	89.79%	0.88%	63.46%	3.22%	68.02%	0.77%	71.87%	1.17%	62.55%	1.31%

c.4253+43G>A (both exon skipping as one event)

Band	WT midigene								Mutant midigene							
	NT		AON1		AON2		SON		NT		AON1		AON2		SON	
	%	SD	%	SD	%	SD	%	SD	%	SD	%	SD	%	SD	%	SD
exon skipping	8.12%	0.38%	14.09%	0.26%	8.53%	0.95%	10.21%	0.88%	36.54%	3.22%	31.98%	0.77%	28.13%	1.17%	37.45%	1.31%
WT	91.88%	0.38%	85.91%	0.26%	91.47%	0.95%	89.79%	0.88%	63.46%	3.22%	68.02%	0.77%	71.87%	1.17%	62.55%	1.31%

Table_S8. Allele frequency, observed RNA, predicted protein defects for six *ABCA4* intronic variants.

Genomic position	Variant	% correctly spliced mRNA based on HEK293T assays	% correctly spliced mRNA based on fibroblast RT-PCR studies#	RNA defect	Protein defect	AC_study cohort (36 cases)	AC_validation cohort (French/German, 412 cases)
g.94549781G>A	c.769-784C>T	91.4	81.8	r.[=, 768_769ins(162)]	p.[=, Leu257Aspfs*3]	7	4
g.94546780C>G	c.859-506G>C	24.4	7.4	r.[858_859ins(56), =]	p.[Phe287Thrfs*32, =]	3	0
g.94527698G>C	c.1937+435C>G	91.37	n.a.	r.[=, 1937_1938ins(134)]	p.[=, Ser646Serfs*25]	2	not tested
g.94496509C>T	c.4253+43G>A	63.46	n.a.	r.[=, 4129_4253del(125)]	p.[=, Ile1377Hisfs*3	9	29
g.94493901T>C	c.4539+1100A>G	19.2	n.a.	r.[4539_4540ins(68), 4539_4540ins(112), =]	p.[Arg1514Valfs*31, Arg1514Glyfs*3, =]	2	0
g.94493895G>A	c.4539+1106C>T	3.02	n.a.	r.[4539_4540ins(68), 4539_4540ins(112)]	p.[Arg1514Valfs*31, Arg1514Glyfs*3]	1	1

AC, allele count; AF, allele frequency; n.a., not applicable; #, mRNA of other allele can also be present in correctly spliced mRNA

Table_S9. Allele frequency comparisons for six *ABCA4* intronic variants in eight alleles in different population databases.

Genomic position	Allele	AC_study cohort (36 cases)	AF in Nijmegen STGD1 cohort (250 cases)	GoNL_AF (in 998 alleles)	Chi-square *p* -value$	gnomAD_nFE_AF
g.94549781G>A	c.769-784C>T	#7	0.0140	0.0080	0.5800	0.004263
g.94549781G>A/g.94476467T>A	c.[769-784C>T; 5603A>T]	7	0.0140	0.0020	0.0230	n.k.
g.94546780C>G	c.859-506G>C	3	0.0060	n.a.	n.a.	n.a.
g.94527698G>C	c.1937+435C>G	2	0.0040	n.a.	n.a.	n.a.
g.94496509C>T	c.4253+43G>A	9	0.0180	0.0050	0.0690	0.005979
g.94496509C>T/g.94471747A>T	[c.4253+43G>A;c.6006-609T>A]	8	0.0160	0.0030	0.0270	n.k.
g.94493901T>C	c.4539+1100A>G	2	0.0040	n.a.	n.a.	n.a.
**Genomic position**	**Allele**	**AC_study cohort (36 cases)**	**AF in UK STGD1 cohort (45 cases)**	**UK10K_AF (in 13,374 alleles)**	**Fischer exact p-value$**	**gnomAD_nFE_AF**
g.94493895G>A	c.4539+1106C>T	1	0.0111	0.0001	0.07000	n.a.

AC, allele count; AF, allele frequency; n.a., not applicable since the region was not covered; n.k., not known; #, this variant was not found without c.5603A>T; $, Bonferroni corrected.

Table_S10. Capillary fragment analyzer results for RT-PCR products of control and STGD1 probands-derived fibroblast harboring c.769-784C>T or c.859-506G>C. Fibroblasts were non-treated (NT) or treated with three different AONs or sense oligonucleotide (AON1, AON2, AON3, SON) in presence (+) or absence (-) of cycloheximide (CHX) treatment. For both variants, AONs were designed to induce pseudoexon exclusion. Two independent experiments were performed and subsequently two RT-PCR per experiment were performed, products were run onto a capillary electrophoresis system to quantify the different fragment quantities expressed as percent (%) of the entire fragment mix. SD, standard deviation.

Pseudoexon exclusion in fibroblasts – fragment analyzer results

c.769-784C>T

Band	Control fibroblasts												Mutant fibroblasts											
	NT -CHX		NT +CHX		AON1		AON2		AON3		SON		NT -CHX		NT +CHX		AON1		AON2		AON3		SON	
	%	SD	%	SD	%	SD	%	SD	%	SD	%	SD	%	SD	%	SD	%	SD	%	SD	%	SD	%	SD
WT	99.53%	0.66%	97.53%	0.40%	94.71%	7.49%	98.89%	0.33%	94.70%	2.39%	97.87%	1.76%	96.81%	4.51%	81.76%	10.43%	43.06%	12.96%	100.00%	0.00%	92.84%	4.65%	49.81%	4.48%
AON-created PE	0.00%	0.00%	0.33%	0.46%	3.57%	5.05%	0.00%	0.00%	0.00%	0.00%	0.00%	0.00%	0.00%	0.00%	0.00%	0.00%	56.39%	12.17%	0.00%	0.00%	0.00%	0.00%	0.00%	0.00%
PE	0.47%	0.66%	2.14%	0.06%	1.72%	2.44%	1.11%	0.33%	5.30%	2.39%	2.13%	1.76%	3.19%	4.51%	18.24%	10.43%	0.56%	0.79%	0.00%	0.00%	7.16%	4.65%	50.19%	4.48%

c.859-506G>C

Band	Control fibroblasts												Mutant fibroblasts											
	NT -CHX		NT +CHX		AON1		AON2		AON3		SON		NT -CHX		NT +CHX		AON1		AON2		AON3		SON	
	%	SD	%	SD	%	SD	%	SD	%	SD	%	SD	%	SD	%	SD	%	SD	%	SD	%	SD	%	SD
WT	100.00%	0.00%	100.00%	0.00%	100.00%	0.00%	100.00%	0.00%	100.00%	0.00%	100.00%	0.00%	100.00%	0.00%	73.38%	10.17%	98.80%	1.70%	41.28%	0.19%	97.41%	3.66%	68.40%	7.06%
PE	0.00%	0.00%	0.00%	0.00%	0.00%	0.00%	0.00%	0.00%	0.00%	0.00%	0.00%	0.00%	0.00%	0.00%	26.62%	10.17%	1.20%	1.70%	58.72%	0.19%	2.59%	3.66%	31.60%	7.06%

Table_S11. Oligonucleotides to introduce mutations in wild-type entry clones

Wild-type fragment	DNA variant_primer orientation	Sequence (5'-3')
BA4	c.768+7329A>G_forward	cccttccctattatctgttcaaactcacatgaattgtttctgtcaaa
BA4	c.768+7329A>G_reverse	tttgacagaaacaattcatgtgagtttgaacagataatagggaaggg
BA6	c.769-784C>T_forward	ctcagtcctacatgactcttctaggatcagtgattcc
BA6	c.769-784C>T_reverse	ggaatcactgatcctagaagagtcatgtaggactgag
BA7	c.858+526T>G_forward	ttgcacaagtctcctcccatagagaaatggattcttcc
BA7	c.858+526T>G_reverse	ggaagaatccatttctctatgggaggagacttgtgcaa
BA7	c.859-506G>C_forward	ctgtgatttgttgttgttgttgttgttttgagacggagtattgctcag
BA7	c.859-506G>C_reverse	gacactaaacaacaacaacaacaacaaaactctgcctcataacgagtc
BA9	c.1555-5008C>T_forward	cacatcttccctagatcaagatagagcaattgtagaagctg
BA9	c.1555-5008C>T_reverse	cagcttctacaattgctctatcttgatctagggaagatgtg
BA11	c.1937+435C>G_forward	tcatggaaatgttagttagcgaaggggctgcattattc
BA11	c.1937+435C>G_reverse	gaataatgcagccccttcgctaactaacatttccatga
BA19	c.4253+43G>A_forward	catgtgacccaggtgtcccaaacccacagag
BA19	c.4253+43G>A_reverse	ctctgtgggtttgggacacctgggtcacatg
BA21	c.4539+1100A>G_forward	cccaggagatgctcaccgtaatctgttctggac
BA21	c.4539+1100A>G_reverse	gtccagaacagattacggtgagcatctcctggg
BA21	c.4539+1106C>T_forward	ctaaatcccccaggagatactcactgtaatctgttct
BA21	c.4539+1106C>T_reverse	agaacagattacagtgagtatctcctgggggatttag
BA30	c.6006-609T>A_forward	ctaatctcatccttttcttcttttagcttagggtctatgaataaag
BA30	c.6006-609T>A_reverse	ctttattcatagaccctaagctaaaagaagaaaaggatgagattag
BA30	c.6148-421T>C_forward	ccgacacatacctgtggagcactgaccatacg
BA30	c.6148-421T>C_reverse	cgtatggtcagtgctccacaggtatgtgtcgg

Table_S12. Primers to analyze mutations introduced in wild-type entry clones

BA_ID	DNA variant	Sequence (5'-3')	Genomic position (hg19)	cDNA position (hg19)	Primer ID
BA4	c.768+7329A>G	TGTTTGCCATTTTGATCTGTGAC	94557129	768+7221	75717
BA6	c.769-784C>T	GGTCCCCAGATACTTGGATG	94549923	769-926	71172
BA7	c.858+526T>G	AAGCACCTGTTCACATCCCA	94548772	858+136	75351
BA7	c.859-506G>C	CCCAAGAACTGGCTTAACAG	94546932	859-658	71174
BA9	c.1555-5008C>T	ACAATAGCCAGTTCTTGTCTACC	94534305	1555-5431	75738
BA11	c.1937+435C>G	TGGTAGTGTGTCTGACCCCAT	94527479	1937+654	59845
BA19	c.4253+43G>A	ACCCTGCTTATTATGTTCCCC	94496836	4129-160	61737
BA21	c.4539+1100A>G	GTTGAACTCCTGGCCTCAAG	94494263	4539+738	74413
BA21	c.4539+1106C>T	GTTGAACTCCTGGCCTCAAG	94494263	4539+738	74413
BA30	c.6006-609T>A	ACCCTACTTGCTTCCCTGAG	94471880	6006-742	71168
BA30	c.6148-421T>C	ACAGCACTGTCACGATCTGC	94468105	6148-557	59856

Table_S13. smMIPs analysed regions

Gene Probe ID	Targeted variant	Targeted variant genomic position	smMIP details (chr:positions/extension seq length, ligation sequence length/strand)	Rank score	Extension probe start	Extension probe stop	Ext probe copy	Extension probe sequence	Ligation probe start	Ligation probe stop	Lig probe copy	Ligation probe sequence	smMIP start position	smMIP stop position	feature_start_position	feature_stop_position	Probe strand	failure flags	Average Coverage
ABCA4_intn_226	c.769-784C>T	94549781	1:94549727-94549876/20,20/+	0,904615	94549727	94549746	1	CCTGTTTGACTTCATGCTTC	94549857	94549876	1	AGTGAGCACTGGGCATGTGC	94549747	94549856	94549481	94550081	+	0	971
ABCA4_intn_119	c.769-784C>T	94549781	1:94549714-94549863/18,22/-	0,90975	94549846	94549863	1	GCTCACTTCACACCTACT	94549714	94549735	1	TCAAACAGGACATGACCTTGGT	94549736	94549845	94549481	94550081	-	0	457
ABCA4_intn_220	c.859-506G>C	94546780	1:94546712-94546861/17,23/+	0,897071	94546712	94546728	14	CAGAGCTTACAGTGAGT	94546839	94546861	1	GCCTCTCTTCTTCTAGTCTCCTT	94546729	94546838	94546530	94547130	+	0	461
ABCA4_intn_112	c.859-506G>C	94546780	1:94546694-94546843/20,20/-	0,830313	94546824	94546843	1	GAGGCTATACTACAGTGCTC	94546694	94546713	13	TGCCCCCCAGGTTCACGCCA	94546714	94546823	94546530	94547130	-	0	179
ABCA4_28_A174	c.4253+43G>A	94496509	1:94496463-94496612/21,19/-	0,904792	94496592	94496612	1	GCGAATACCCCGCTTTGACCC	94496463	94496481	1	GCCCTGACCTTCCCACTTC	94496482	94496591	94496525	94496701	-	10	362
ABCA4_28_S175	c.4253+43G>A	94496509	1:94496448-94496597/21,19/+	0,833598	94496448	94496468	1	GGTGAAGGTCCCAGTGAAGTG	94496579	94496597	1	ATCCAGGGGTGAAGGGTCA	94496469	94496578	94496525	94496701	+	0	151
ABCA4_intn_178	c.4539+1100A>G	94493901	1:94493867-94494016/16,24/+	0,744014	94493867	94493882	6	TTTGATGTCTCTAAAT	94493993	94494016	1	GCATGTATTTGTGTACATACCTGC	94493883	94493992	94493595	94494201	+	0	176
ABCA4_intn_65	c.4539+1100A>G	94493901	1:94493828-94493977/16,24/-	0,839159	94493962	94493977	7	CTTTTGCAGGGACATT	94493828	94493851	1	GGTTGATAATGGGCCTCCCAGCTG	94493852	94493961	94493595	94494201	-	0	535
ABCA4_intn_178	c.4539+1106C>T	94493895	1:94493867-94494016/16,24/+	0,744014	94493867	94493882	6	TTTGATGTCTCTAAAT	94493993	94494016	1	GCATGTATTTGTGTACATACCTGC	94493883	94493992	94493595	94494201	+	0	176
ABCA4_intn_65	c.4539+1106C>T	94493895	1:94493828-94493977/16,24/-	0,839159	94493962	94493977	7	CTTTTGCAGGGACATT	94493828	94493851	1	GGTTGATAATGGGCCTCCCAGCTG	94493852	94493961	94493595	94494201	-	0	535

Gene Probe ID	Targeted variant	Target sequence
ABCA4_intn_226	c.769-784C>T	ATGTTTCAAAACTGATGGAATCACTGATCCTAGAGGAGTCATGTAGGACTGAGTTCTAAGTGTAAAGACAGTGGTTTTCCAACTCAAGTGTGCTTCCTGAGTAGGTGTGA
ABCA4_intn_119	c.769-784C>T	CAGGAAGCACACTTGAGTTGGAAAACCACTGTCTTTACACTTAGAACTCAGTCCTACATGACTCCTCTAGGATCAGTGATTCCATCAGTTTTGAAACATGAAGCATGAAG
ABCA4_intn_220	c.859-506G>C	AGACATCGTGCCACTGCACTCCAGCCTGGGCGACTGAGCAATACTCCGTCTCAAAACAACAACAACAACAACAACAAATCACAGAGTGACCCCTAGAGCACTGTAGTATA
ABCA4_intn_112	c.859-506G>C	TAGGGGTCACTCTGTGATTTGTTGTTGTTGTTGTTGTTTTGAGACGGAGTATTGCTCAGTCGCCCAGGCTGGAGTGCAGTGGCACGATGTCTACTCACTGTAAGCTC
ABCA4_28_A174	c.4253+43G>A	TTCACCCCTGGATATATGGGCAGCAGTACACCTTCTTCAGGTGCGCGGACTCGGGGTCACCATTCTCCTCTGTGGGTTTGGGGCACCTGGGTCACATGCTGCTTAGAAGG
ABCA4_28_S175	c.4253+43G>A	GGAAGGTCAGGGCCCTTCTAAGCAGCATGTGACCCAGGTGCCCCAAACCCACAGAGGAGAATGGTGACCCCGAGTCCGCGCACCTGAAGAAGGTGTACTGCTGCCCATAT
ABCA4_intn_178	c.4539+1100A>G	CCCCCAGGAGATGCTCACTGTAATCTGTTCTGGACTTTAGAACTCCCAGGACAGGGTGCTGCTCAGAGCCAAGATCATTAATGTCCCTGCAAAAGATGGATGCTGGCTGT
ABCA4_intn_65	c.4539+1100A>G	AATGATCTTGGCTCTGAGCAGCACCCTGTCCTGGGAGTTCTAAAGTCCAGAACAGATTACAGTGAGCATCTCCTGGGGGATTTAGAGACATCAAAGAAGGCTGTGTCCGT
ABCA4_intn_178	c.4539+1106C>T	CCCCCAGGAGATGCTCACTGTAATCTGTTCTGGACTTTAGAACTCCCAGGACAGGGTGCTGCTCAGAGCCAAGATCATTAATGTCCCTGCAAAAGATGGATGCTGGCTGT
ABCA4_intn_65	c.4539+1106C>T	AATGATCTTGGCTCTGAGCAGCACCCTGTCCTGGGAGTTCTAAAGTCCAGAACAGATTACAGTGAGCATCTCCTGGGGGATTTAGAGACATCAAAGAAGGCTGTGTCCGT

Gene Probe ID	Targeted variant	smMIP sequence
ABCA4_intn_226	c.769-784C>T	AGTGAGCACTGGGCATGTGCNNNNNNNNCTTCAGCTTCCCGATATCCGACGGTAGTGTCCTGTTTGACTTCATGCTTC
ABCA4_intn_119	c.769-784C>T	TCAAACAGGACATGACCTTGGTNNNNNNNNCTTCAGCTTCCCGATATCCGACGGTAGTGTGCTCACTTCACACCTACT
ABCA4_intn_220	c.859-506G>C	GCCTCTCTTCTTCTAGTCTCCTTNNNNNNNNCTTCAGCTTCCCGATATCCGACGGTAGTGTCAGAGCTTACAGTGAGT
ABCA4_intn_112	c.859-506G>C	TGCCCCCCAGGTTCACGCCANNNNNNNNCTTCAGCTTCCCGATATCCGACGGTAGTGTGAGGCTATACTACAGTGCTC
ABCA4_28_A174	c.4253+43G>A	GCCCTGACCTTCCCACTTCNNNNNNNNCTTCAGCTTCCCGATATCCGACGGTAGTGTGCGAATACCCCGCTTTGACCC
ABCA4_28_S175	c.4253+43G>A	ATCCAGGGGTGAAGGGTCANNNNNNNNCTTCAGCTTCCCGATATCCGACGGTAGTGTGGTGAAGGTCCCAGTGAAGTG
ABCA4_intn_178	c.4539+1100A>G	GCATGTATTTGTGTACATACCTGCNNNNNNNNCTTCAGCTTCCCGATATCCGACGGTAGTGTTTGATGTCTCTAAAT
ABCA4_intn_65	c.4539+1100A>G	GGTTGATAATGGGCCTCCCAGCTGNNNNNNNNCTTCAGCTTCCCGATATCCGACGGTAGTGTCTTTTGCAGGGACATT
ABCA4_intn_178	c.4539+1106C>T	GCATGTATTTGTGTACATACCTGCNNNNNNNNCTTCAGCTTCCCGATATCCGACGGTAGTGTTTGATGTCTCTAAAT
ABCA4_intn_65	c.4539+1106C>T	GGTTGATAATGGGCCTCCCAGCTGNNNNNNNNCTTCAGCTTCCCGATATCCGACGGTAGTGTCTTTTGCAGGGACATT
